# Haemosporidian parasite infections of Malagasy Philepittidae and Nectariniidae are driven by phylogeny rather than ecology

**DOI:** 10.1017/S0031182023001075

**Published:** 2023-12

**Authors:** Hannah Barbon, Jean-Louis Berthoud, Friederike Woog, Sandrine Musa

**Affiliations:** 1 Department of Parasitology, University of Hohenheim, Stuttgart, Germany; 2 State Museum of Natural History Stuttgart, Stuttgart, Germany

**Keywords:** *Cinnyris* spp, filarioid nematodes, *Haemoproteus*, *Leucocytozoon*, Nectariniidae, *Neodrepanis coruscans*, *Philepitta castanea*, *Plasmodium*, *Trypanosoma*

## Abstract

The nectarivorous common sunbird asity (*Neodrepanis coruscans*) is phylogenetically closely related to the frugivorous velvet asity (*Philepitta castanea*), yet it shares similar habitat and foraging behaviour as the Malagasy sunbirds (*Cinnyris* spp.). As ecological factors have been shown to influence blood parasite prevalence, it should be tested whether parasite abundance, prevalence and diversity of *N. coruscans* are more similar to the sunbirds than to its relative. Therefore, blood samples (*n* = 156) and smears (*n* = 60) were tested for different blood parasites (Haemosporida, trypanosomes, filarioid nematodes) using molecular and microscopic methods. High prevalence of haemosporidian parasites was observed in all bird taxa, with rates ranging from 23% in *N. coruscans* to 84.6% in *C. notatus*. The Malagasy *Cinnyris* spp. exhibited a high occurrence of mixed haemosporidian infections (>76%) with various specialized lineages. Within the Philepittidae family, no *Haemoproteus* infection was detected and just a few cases of mixed infections. Nectariniidae species predominantly had specialized haemosporidian lineages, while Philepittidae had infections mainly caused by generalist lineages. These findings emphasize the diverse range of blood parasites in Nectariniidae, while additionally highlighting the high diversity of trypanosomes and filarioid nematodes in Philepittidae. Additionally, several newly discovered haemosporidian lineages, *Trypanosoma* isolates and filarioid nematode isolates were identified. Notably, Philepittidae exhibited a lower prevalence of avian haemosporidian parasites compared to Nectariniidae, possibly due to potential resistance mechanisms. Despite *N. coruscans* sharing similar habitat and behavioural ecology with both *Cinnyris* spp., it closely resembles its relative, *P. castanea*, in all aspects of haemosporidian parasitism.

## Graphical abstract

**Figure:**
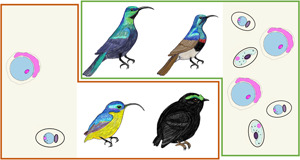

## Introduction

**Figure 1.**
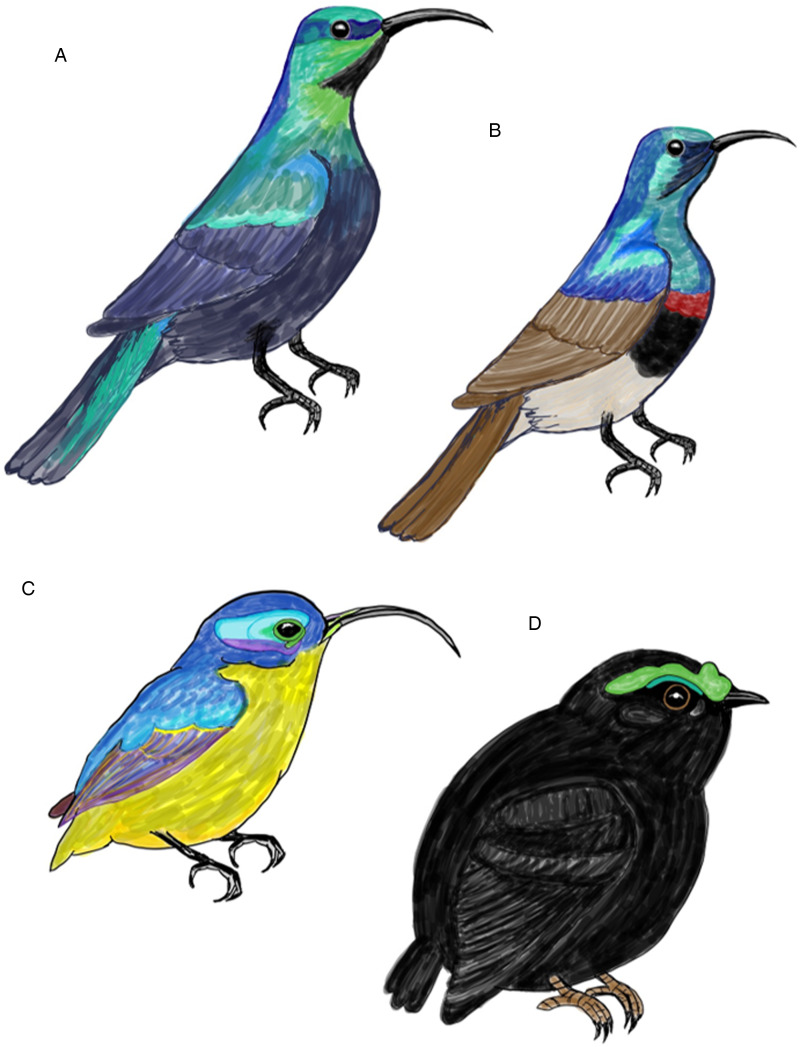
Illustrations of breeding males of examined Malagasy bird species: (a) Madagascar green sunbird (*Cinnyris notatus*), (b) souimanga sunbird (*Cinnyris sovimanga*), (c) common sunbird asity (*Neodrepanis coruscans*), (d) velvet asity (*Philepitta castanea*). Females have inconspicuous colorations in comparison. Drawings by S. Musa.

The asities (Philepittidae) are a family of old world suboscine birds of Madagascar and form a single radiation of only 4 extant species (Goodman, 2022). Their ancestors likely arrived from Asia to Madagascar approximately 41 million years ago (Warren *et al*., 2010). These 4 endemic species are divided into 2 different genera (*Neodrepanis* spp. and *Philepitta* spp.), which exhibit significant differences in their physical characteristics and behavioural ecology. *Neodrepanis* are nectar-feeding birds characterized by their slender bill. On the other hand, *Philepitta* species have shorter beaks (Fig. 1) and are among the few primary frugivorous songbirds in Madagascar. All 4 species exhibit sexual dimorphism in terms of plumage and physical features. The Schlegel's asity (*Philepitta schlegeli*) is primarily found, even if scarce, in the western forests of Madagascar. In contrast, the velvet asity (*Philepitta castanea*) inhabits eastern rainforests at altitudes below 1500 m (Hawkins *et al*., 2015). It tends to be sedentary and is thought to play a role as a seed disperser, primarily foraging on small fruits in the understory shrubs at heights ranging from 2.5 to 5 m. Additionally, they construct elongated globe-shaped nests, typically positioned 2–7 m above the ground (Safford and Hawkins, 2013). *Neodrepanis* asities were formerly categorized as sunbirds (Nectariniidae) due to the similarities in ecological traits and physical characteristics. One of these species, the yellow-bellied sunbird asity (*N. hypoxantha*), is found exclusively in high-altitude humid forests between 1200 and 2500 m in eastern Madagascar. Whereas the common sunbird asity (*N. coruscans*) occurs in mid-altitude rainforests, typically occupying elevations ranging from 500 to 1500 m. It is a small and exceptionally active bird that inhabits the canopy and subcanopy layers. It is often seen accompanying mixed-species flocks (Eguchi *et al*., 1993) and, occasionally, it competes with the souimanga sunbird (*Cinnyris sovimanga*) for access to flowers (Safford and Hawkins, 2013). The construction of the nests is similar to that of the velvet asity, featuring a small entrance hole and suspended from vegetation, although typically positioned somewhat higher, around 5–8 m above the ground (Safford and Hawkins, 2013).

The 2 sunbird species endemic to Madagascar occupy similar habitat and behavioural ecology as the *Neodrepanis* species. In contrast to the asities, sunbirds belong to the oscines and represent a relatively recent radiation, dating back approximately 3.9 million years. It appears that the 2 species arrived on Madagascar through 2 separate colonization events originating from the African continent. They used Indian Ocean islands like the Comoros as stepping stones during their journey from Africa (Warren *et al*., 2003). The souimanga sunbird (*C. sovimanga*) is abundantly found in all wooded habitats across Madagascar (Hawkins *et al*., 2015) and often joins mixed species flocks (Eguchi *et al*., 1993). It utilizes various vegetation levels, ranging from the canopy of tall forests to shrubbery in clearings, and scattered areas of trees and bushes in open ground (Safford and Hawkins, 2013). The Madagascar green sunbird (*Cinnyris notatus*) is also distributed throughout Madagascar, with its presence extending up to elevations of 2050 m; however, in forests, it prefers to forage mid-stratum and in the canopy (Hawkins *et al*., 2015). It can be distinguished from the souimanga sunbird and sunbird asities by its long, strong bill, a larger size and darker plumage of the male (Fig. 1). Both sunbird species show active and restless behaviour. They primarily feed on nectar, but they also capture spiders and insects from flowers. Their strong bills enable them to feed on larger flowers, while flowers with long corollas remain inaccessible to the souimanga sunbird (Safford and Hawkins, 2013). In terms of sexual dimorphism, both species exhibit distinct differences between males and females regarding their plumage and morphology. Females in both species engage in nest construction and incubation, while males are either absent or responsible for defending their territory. The nests built by both species share a design typical for sunbirds, featuring a domed structure with a side entrance. However, the height of these nests varies, with souimanga sunbirds typically placing their nests at 0.5–1.8 m above the ground, while Madagascar green sunbird species opt for higher nest locations (Safford and Hawkins, 2013).

Previous studies highlighted that various ecological traits and the geographical distribution of birds exert a significant influence on the abundance, prevalence and diversity of parasites. This pertains specifically to parasites such as protists belonging to the order Haemosporida (Apicomplexa), the genus *Trypanosoma* (Euglenozoa) and metazoa like filarioid nematodes, all of which share a common habitat at some stage of their life cycles – the bloodstream. Among these parasites, the haemosporidian genera (*Plasmodium*, *Haemoproteus* and *Leucocytozoon*) develop their gametocytes within the blood cells of their avian hosts. Notably, *Plasmodium* is the sole genus that also undergoes a phase of asexual reproduction within erythrocytes (Valkiunas, 2005). In contrast, Trypanosomes, while not entirely intracellular parasites like the Haemosporida, reside within the bloodstream or the intestines of their host. Only the amastigote form of the parasite is intracellular (Moretti *et al*., 2020). Parasitic nematodes, on the other hand, primarily infect host tissues and tissue spaces, with their larval stages occasionally appearing in peripheral blood circulation (Sehgal *et al*., 2005). All blood parasites play pivotal roles as avian parasites, and exhibit a global distribution and transmission *via* a variety of blood-sucking insects. They often co-occur in mixed infections (Savage *et al*., 2009) and in most instances, they do not pose significant harm to their avian hosts. However, it is important to acknowledge that research efforts have been disproportionately allocated among different parasite taxa, with the majority of available data focusing on *Plasmodium* and *Haemoproteus*, primarily due to their close relationship with agents responsible for human malaria (Atkinson *et al*., 2008; Santiago-Alarcon and Marzal, 2020).

The abundance, prevalence and diversity of parasites in different bird species are influenced by various factors, including the extent of host specialization. Specialization plays a crucial role in determining which parasites are capable of infecting a particular host species. Parasites that exhibit a high degree of specialization, focusing on their specific bird hosts, tend to have a narrower host range and consequently a more limited geographic distribution. A case in point is the highly specialized haemosporidian lineages found in Vangas (Vangidae), which are confined to the geographic range of their hosts, primarily Madagascar (Magaña Vázquez *et al*., 2022). In contrast, generalist avian malaria parasites like *Plasmodium relictum* are characterized by a global distribution and a remarkable ability to invade diverse host species (Hellgren *et al*., 2015). Avian *Trypanosoma* parasites, on the other hand, do not exhibit strict vertebrate–host specificity (Valkiunas *et al*., 2011), while filarioid nematodes are believed to be highly specialized for their avian hosts (Binkienė *et al*., 2021). Within the realm of avian Haemosporida parasites, the degree of specialization varies not only between host genera but also among different species within the same genus. In general, *Plasmodium* and *Leucocytozoon* species are often considered to be more generalists, capable of infecting a broader range of hosts, whereas *Haemoproteus* species tend to exhibit a high degree of specialization in most instances. This specialization pattern has been observed in various studies (e.g. Musa *et al*., 2019; Doussang *et al*., 2021).

Previous research has demonstrated that the diversity of haemosporidian parasites is closely linked to the richness of avian species (Clark *et al*., 2014) and that the specific assembly of parasite communities is largely influenced by the composition of their host species (Fecchio *et al*., 2019). However, the likelihood of birds becoming infected with blood parasites depends on their interaction with the vectors, and this connection is strongly influenced by the ecological characteristics and distribution of these vectors. For instance, in regions with high elevations, there is a notable prevalence of black flies (Simuliidae), which serve as vectors for *Leucocytozoon* spp. In contrast, areas at lower elevations tend to have a higher abundance of mosquitoes (Culicidae). This altitudinal variation also extends to the distribution of blood parasites. Therefore, *Leucocytozoon* is more prevalent at higher elevations, followed by *Haemoproteus* and *Plasmodium* (Rodríguez-Hernández *et al*., 2021), whereas trypanosomes and filarioid nematodes are predominantly found at lower elevation areas (Chapa-Vargas *et al*., 2020). Ornithophilic mosquitoes exhibit greater diversity at the canopy level compared to the forest floor (Chathuranga *et al*., 2022) which may indicate a higher *Plasmodium* diversity in the canopy.

Moreover, recent research has identified several ecological and individual traits that play a significant role in affecting the prevalence of haemosporidian parasites. Incidence of infection is directly correlated to the age and sex of birds (Valkiunas, 2005). Furthermore, it has been observed that foraging behaviour exerts an influence on prevalence rates. In particular, the highest prevalence of *Plasmodium* was found among frugivores, granivores and omnivores, while insectivores exhibited the lowest prevalence (Rodríguez-Hernández *et al*., 2021). On the other hand, *Haemoproteus* prevalence was highest among granivores, insectivores and nectarivores, and *Leucocytozoon* was predominantly present in granivorous and insectivorous birds in Mexico (Rodríguez-Hernández *et al*., 2021). In open nests, it was noted that the prevalence of *Haemoproteus* spp. and *Leucocytozoon* spp. was higher compared to closed nests (Rodríguez-Hernández *et al*., 2021). This difference in prevalence was attributed to the better protection provided by closed nests for both nestlings and breeding adults (Valkiunas, 2005).

The primary hypothesis investigated in this study posited that the blood parasite abundance, prevalence and diversity in the common sunbird asity (*Neodrepanis coruscans*) is akin to that of other nectarivorous bird species in Madagascar, specifically the sunbirds (*C. notatus* and *C. sovimanga*), due to their similar habitat and behavioural ecology. In contrast, the velvet asity (*P. castanea*), a relative of the common sunbird asity, is expected to exhibit differing infection rates. Furthermore, it was tested whether (1) the nectarivorous species have lower prevalence for *Plasmodium* species but a higher diversity in contrast to the frugivorous *P. castanea*, (2) Nectariniidae and Philepitiidae do have their own specialized *Haemoproteus* and filarioid nematode species and (3) due to their breeding in closed nests, the prevalence of *Haemoproteus* and *Leucocytozoon* of both bird taxa will be lower than in other species at that study site using open cups.

## Materials and methods

### Study site and test material

Birds of the family Nectariniidae and Philepittidae were captured in the Maromizaha tropical rainforest located in the eastern part of Madagascar (18580800 S, 482704800 E), 30 km from the city of Moramanga, at 5 different sampling sites with an altitude between 943 and 1213 m. In the course of a ringing project, birds were caught in mist nets mostly during the breeding season in November and December (2003–2007, 2010, 2012, 2014, 2016, 2018 and 2022). Individual data of birds such as age, sex or weight were measured whenever possible. Birds in juvenile plumage were aged as juveniles until having completed post-juvenile moult. This plumage differs in its colouration and structure. In addition, juveniles often displayed some rest of a gape. Birds lacking juvenile characteristics were categorized as adults, with no further opportunity for a more precise determination of their age.

Before release, a tiny drop of blood was taken by puncturing the brachial vein. The protocol was approved by the Direction de la Préservation de la Biodiversité, Antananarivo, Madagascar. Blood was immediately stored in lysis buffer (Wink, 2006) and, if possible, 1–3 blood smears per bird were prepared before the bird was released back into the wild. A total of 82 blood samples of Nectariniidae (*C. sovimanga*, *n* = 56; *C. notatus*, *n* = 13) and 74 Philepittidae (*P. castanea*, *n* = 61; *N. coruscans*, *n* = 13) were collected. Blood smears were prepared for 29 Nectariniidae (*C.is sovimanga*, *n* = 26; *C. notatus*, *n* = 3) and 31 Philepittidae species (*P. castanea*, *n* = 26; *N. coruscans*, *n* = 5). The slides were dried on site and fixed with >99% methanol for 10 min in the field. Blood smears from 2022 were Giemsa stained on site using the Hemacolor® staining set (Merck KGaA, Darmstadt, Germany) following the manufacturer's protocol, blood smears of other years were stained in the lab with the same staining set.

### Microscopic examination of blood smears

Every slide was examined using an AxioImager M2 (Carl Zeiss AG, Oberkochen, Germany). At first, slides were screened at 400 magnification for 10 min to quickly detect larger parasites (*Leucocytozoon* spp., trypanosomes and filarioid nematodes) and then 20 min under high magnification (100 oil-immersion objective, 10 ocular) to detect smaller ones (*Plasmodium* and *Haemoproteus* spp.). Pictures were taken and edited with Zen software (Carl Zeiss AG).

### DNA extraction and molecular detection methods of blood parasites

Total DNA extraction of blood samples was performed using the QIAamp DNA Blood Mini Kit (QIAGEN, Hilden, Germany) following the manufacturer's instructions. DNA concentration and purity were quantified using a NanoDrop N50 UV-Vis spectrophotometer (Implen GmbH, Munich, Germany) and stored at –20°C until further use. Molecular parasite detection was performed using different polymerase chain reaction (PCR) setups. In each test run, a positive control as well as a negative control (nuclease-free water) were included.

#### Haemosporidian parasites

Detection of haemosporidian infections was done by applying 2 different PCR setups. At first, all samples were screened using a nested PCR targeting a 479 bp region of the cytochrome b gene (cytb) of the 3 parasite genera (Hellgren *et al*., 2004) following the protocol described by Magaña Vázquez *et al*. (2022). Samples that showed no bands in the agarose gel were redone a second time. As this nested PCR approach is not able to detect mixed infections with *Plasmodium* and *Haemoproteus*, a second PCR setup was performed for all samples that showed positive signals for either *Plasmodium* or *Haemoproteus* infection. This PCR setup was a nested multiplex PCR which detects lineages of *Plasmodium* and *Haemoproteus* parasites separately, by yielding amplicons with different sizes (Pacheco *et al*., 2018). PCR reactions of the primary PCR were carried out in a total volume of 25 *μ*L, containing 2.5 *μ*L GeneAmp™ 10× PCR Buffer II (Applied Biosystems, Carlsbad, CA, USA), 2 *μ*L MgCl_2_ (25 mm), 1 *μ*L of each primer (AE298/AE299; 10 mm), 0.5 *μ*L of each dNTP (10 mol), 0.125 *μ*L AmpliTaq™ DNA polymerase (5 U *μ*L^−1^; Applied Biosystems), 2 *μ*L template DNA (10–100 ng *μ*L^−1^) and 15.875 *μ*L nuclease-free water. The reaction mixture of the nested multiplex PCRs consisted of 5 *μ*L GeneAmp™ 10× PCR Buffer II (Applied Biosystems), 4 *μ*L MgCl_2_ (25 mm), 2 *μ*L of each primer (AE980/AE982 and AE983/AE985; 10 mm), 1 *μ*L of each dNTP (10 mol), 0.25 *μ*L AmpliTaq™ DNA polymerase (5 U *μ*L^−1^; Applied Biosystems), 2 *μ*L amplification product of the initial PCR and 33.75 *μ*L nuclease-free water in a total volume of 50 *μ*L. Cycling conditions of both PCRs were performed as described by Pacheco *et al*. (2018). Amplification products (10 *μ*L) of the nested multiplex PCR were mixed with GelRed™ stain (BIOTREND, Köln, Germany) and visualized on a 2% agarose gel after 30 min at 90 V. DNA fragments of 580 bp length belong to *Plasmodium* spp. whereas fragments of 346 bp length are considered to be *Haemoproteus* species.

#### Trypanosomes

Presence of *Trypanosoma* DNA was detected by nested PCR targeting a 770 bp SSU rRNA fragment (Valkiunas *et al*., 2011). Reaction mixtures were equal to the nested PCR used for haemosporidian detection. The first set of primers was Tryp763 and Tryp1016 and the second pair was Tryp99 and Tryp957. Cycling conditions of both PCRs were performed as described by Valkiunas *et al*. (2011). Amplification products (5 *μ*L) of the nested PCR were also mixed with GelRed™ stain and then visualized on a 1.5% agarose gel after 20 min at 90 V.

#### Filarioid nematodes

Screening for microfilariae was performed using a nested PCR assay targeting an approximately 690 bp fragment of the 28S rRNA. Reaction mixtures were equal to the nested PCR used for haemosporidian and *Trypanosoma* detection. The first set of primers was 28SNemF1 and 28SNemR1 and the second set was 28SNemF2 and 28SNemR2. Cycling conditions of both PCRs were performed as described by Magaña Vázquez *et al*. (2022). A second nested PCR, targeting a 650 bp fragment of the cox1 gene, was used for samples where DNA amplification of the microfilarial 28S rRNA fragment was successful. Reaction mixtures were equal to the nested PCR targeting the microfilarial 28S rRNA, beside the use of the specific primer set COINemF1/COINemR1 and COINemF2/COINemR2, and cycling conditions were also performed after Magaña Vázquez *et al*. (2022).

### Sequence identification

All amplification products, except those of the nested multiplex PCR, were purified using the PCR Product Purification Kit (Roche, Mannheim, Germany). After sequencing (Microsynth AG, Balgach, Switzerland), the resulting sequence data were checked and edited using Geneious v. 2021.1.1 (https://www.geneious.com). If the chromatogram showed clear double peaks, the sample was considered to contain a mixed infection. The final sequences were then distinguished by identifying their closest matches in GenBank (Benson *et al*., 2013) using the NCBI nucleotide BLAST search. Avian haemosporidian parasites were additionally identified using the BLAST search of the MalAvi database (Bensch *et al*., 2009). If haemosporidian sequences differed in at least 1 base pair, it was considered a new lineage and naming was performed according to the system used for the MalAvi database: the first 3 letters of the bird genus, the first 3 letters of the species name, followed by a number (e.g. CINNOT03 from *C. notatus*). In the text, those lineage names were always accompanied by an abbreviation of its parasite genus (p: *Plasmodium*, h: *Haemoproteus*, l: *Leucocytozoon*). Newly amplified sequences of trypanosomes and filarioid nematodes were named according to their closest match in GenBank and given an isolate-abbreviation for easier identification. All newly detected parasite lineages/sequences were deposited in GenBank (accession numbers OQ995051–OQ995057, OR148589–OR148300, OR149496–149504, OR159320–OR159331; Supplementary Tables 1–3).

### Morphological and molecular-based sexing

Sexing based on morphology was only deemed trustworthy for adult males in breeding plumage and for females presenting a clear brood patch (*N* = 62). The sex of all other samples was determined using a molecular methodology (*N* = 74) of Díaz Casana *et al*. (2019). As a basis of comparison for the PCR approach, 2 samples for which sexing based on morphology was certain were randomly chosen for both sexes in each species (*N* = 8). PCR reactions were carried out in a total volume of 25 *μ*L, containing 2.5 *μ*L GeneAmp™ 10× PCR Buffer II (Applied Biosystems), 2 *μ*L MgCl_2_ (25 mm), 1 *μ*L of each primer (HPF/HPR; 10 mm), 0.5 *μ*L of each dNTP (10 mol), 0.125 *μ*L AmpliTaq™ DNA polymerase (5 U L^−1^; Applied Biosystems), 2 *μ*L template DNA (1–5 ng *μ*L^−1^) and 15.875 *μ*L nuclease-free water. Cycling conditions were performed after Díaz Casana *et al*. (2019). Amplification products (10 *μ*L) were mixed with GelRed™ stain and visualized on a 2% agarose gel after 30 min at 90 V. The PCR products in female individuals should show 2 bands, the W band (350–450 bp) and the Z band (500–600 bp), while males show a single band (500–600 bp).

### Phylogenetic analyses

Phylogenetic analyses of haemosporidian lineages, trypanosomes and filarioid nematodes detected in this study were performed using MEGA v.10.2 (Kumar *et al*., 2018). The dataset used for the first phylogenetic approach consisted of cytochrome b sequences of haemosporidian lineages obtained in this study and reference sequences of different morphospecies of Haemosporida downloaded from GenBank, each trimmed to 453 bp to ensure consistency in sequence length. A cytochrome *b* sequence from *Leucocytozoon grallariae* (MK103895) was used as an outgroup. The second tree included all 18S rRNA sequences of *Trypanosoma* detected in the sample set and homologous *Trypanosoma* sequences from GenBank, each trimmed to 669 bp. The human pathogen *Trypanosoma brucei* (AF306777) was used as an outgroup. The third and fourth dataset consisted of all sequences of microfilariae detected in this study. Partial sequences of cox1 (571) and 28S rRNA (681) were used to perform separate phylogenetic analyses. *Ascaridia galli* (KY990014 and KT613888) was used as an outgroup in both approaches. Phylogenies were generated by implementing the best-fitting model, which was identified by MEGA v.10.2 for every parasite taxon. All maximum likelihood methods were performed using 1000 replicates. The resulting phylograms were viewed and edited with MEGA v.10.2.

### Statistical analyses

Significance of detected differences in prevalence of blood parasite infection between the different bird taxa was measured using the *χ*^2^-test implemented in R-4.2.1. The influence of age and sex of the birds was also tested with the *χ*^2^-test.

### Degree of specialization

The study utilized the host-diversity index developed by Musa *et al*. (2019) to assess the level of specialization exhibited by each haemosporidian parasite lineage. The aim was to determine whether specialization occurred at the species, genus or family level, or if the lineages demonstrated a more generalized pattern. This index assigns a value of 1 to indicate maximum host diversity and 0 to signify minimal host diversity. In the context of parasitism, generalist parasite species are capable of infecting a wide range of host taxa, while specialists have a more limited set of potential host taxa. Consequently, parasite species with a high Hd value (>0.6) were categorized as generalists, while those with a value closer to 0 (<0.3) were considered specialists. To arrive at these conclusions, data from the present study and previous research (sourced from MalAvi in June 2023) were compiled. Furthermore, the Hd index was cross-referenced with information from the phylogenetic analysis to provide comprehensive insights into the degree of specialization.

## Results

### Haemosporidian abundance, prevalence and diversity

High prevalence of haemosporidian parasites was observed in all bird taxa, with rates ranging from 23% in *N. coruscans* to 84.6% in *C. notatus* (Table 1). Nectariniidae and *N. coruscans* differed significantly in prevalence (*χ*^2^ = 19.89, d.f. = 1, *P* < 0.001), while both Philepittidae species were more similar in haemosporidian prevalence (*χ*^2^ = 2.96, d.f. = 1, *P* = 0.086). Highest prevalence for all 3 haemosporidian genera was found in *C. notatus* (*Plasmodium*: 61.54%, *Haemoproteus*: 69.23% and *Leucocytozoon*: 84.62%). Lineages of all 3 haemosporidian genera were detected in Nectariniidae while *Haemoproteus* lineages were absent in both Philepittidae species and, in addition, no *Plasmodium* lineages were found in *N. coruscans*.

**Table 1.** Dataset of Nectariniidae and Philepittidae species examined in this study

			Haemosporida			*Trypanosoma*			Filarioid nematode		
Bird species	Nbs	*N*	Pmic	Pmol	Lineages (*n*)	Pmic	Pmol	Sequence (*n*)	Pmic	Pmol	Sequence (*n*)
**Nectariniidae**											
*Cinnyris notatus*	3	13	100	84.6	**pCINNOT03**, pCOSUN2, hCINNOT01 (2), hNENOT04 (5), **lCINNOT02**, lCINSOV02, lCOSUN1 (7)	0	8	–	0	31	***Splendidofilaria* mavis GRESU**, ***Dirofilaria* sp. GRESU**, ***Eufilaria* sp. GRESU**, ***Aproctella alessandroi* GRESU**
*Cinnyris sovimanga*	26	69	87	81.2	pCINCOQ01, pGRW09 (2), pFOUMAD03, pFOUSEY01, pRECOB4, hCINSOV01 (28), **hCINSOV07**, hNENOT04 (4), hXANZOS01, lANLAT10, lCINSOV02 (2), **lCINSOV03** (5), **lCINSOV04**, **lCINSOV05** (2), **lCINSOV06**, **lCINSOV08**, lFOMAD01 (23), lHYPMA02 (2),	0	17	*Trypanosoma* sp. LZ-2011 (JN006849) (2), *Trypanosoma* sp. CORVOID01 (OP006593), ***Trypanosoma anguiformis* SOSU1**, ***Trypanosoma anguiformis* SOSU2**, ***Trypanosoma avium* SOSU**, ***Trypanosoma* sp. SOSU1**, ***Trypanosoma* sp. SOSU2**, ***Trypanosoma* sp. SOSU3**	0	6	***Onchocercidae* sp. SOSU1**, ***Onchocercidae* sp. SOSU2**, ***Aproctella alessandroi* SOSU**, ***Splendidofilaria* sp. SOSU**
Total Nectariniidae	29	82	79.3	81.7		0	15.9		0	9.8	
**Philepittidae**											
*Neodrepanis coruscans*	5	13	20	23	lFOMAD01, lHYPMA02 (2)	0	7.7	***Trypanosoma anguiformis* WATAS**	0	7.7	***Onchocercidae* sp. WATAS**
*Philepitta castanea*	26	61	26.9	49.2	pGRW04 (4), pGRW09 (6), pNEWAM07, lFOMAD01 (4), lHYPMA02 (10), lPHICAS01 (6)	0	11.5	*Trypanosoma* sp. CORVOID01 (OP006593) (3), ***Trypanosoma* sp. VELAS**, ***Trypanosoma bennetti* VELAS**	3.8	13.1	***Splendidofilaria bartletti* VELAS1**, ***Splendidofilaria bartletti* VELAS2**, ***Splendidofilaria bartletti* VELAS3** (2), ***Madathamugadia* sp. VELAS**, ***Splendidofilaria* sp. VELAS1**, ***Splendidofilaria* sp. VELAS2**, ***Chandlerella* sp. VELAS**
Total Philepittidae	31	74	25.8	44.6		0	10.8		3.2	12.2	

Absolute numbers of blood smears (Nbs) and blood samples (N) are given. Results of parasite detections are listed for haemosporidian parasites, trypanosomes and filarioid nematodes, respectively. For each parasite taxon, percentages (%) of infected samples identified with microscopic methods (Pmic) and molecular methods (Pmol), along with the name of the identified lineage/sequence, are given. ‘–’ indicates that percentages were not measurable due to a lack of samples or that no sequence was amplified. If the parasite lineage/sequence was detected more than once, sample sizes are given in brackets. Newly detected lineages/sequences are given in bold letters.

**Figure 2.**
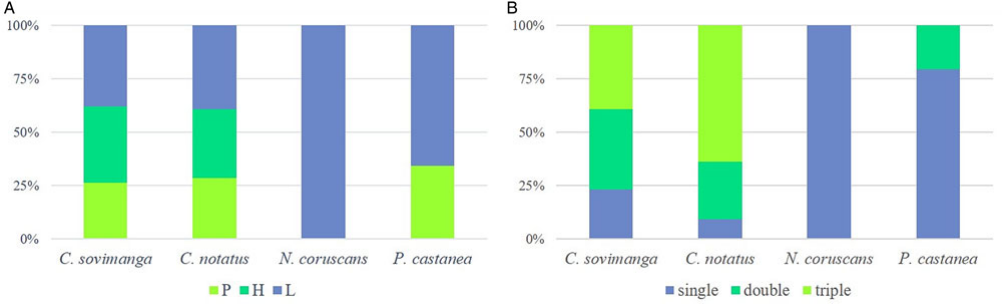
Percentage of shared (a) haemosporidian genera within infected Nectariniidae (*Cinnyris sovimanga* and *C. notatus*) and Philepittidae species (*Neodrepanis coruscans* and *Philepitta castanea*) and percentage of the kind of infections (b) present.

Mixed haemosporidian infections were highly abundant in *C. notatus* (90.9%) and *C. sovimanga* (76.8%) whereas in *P. castanea* few mixed haemosporidian infections were detected (20.7%) and none in *N. coruscans* (Table 2 and Fig. 2).

**Table 2.** Kind of haemosporidian infection (*n*) detected in avian blood samples of Nectariniidae *(Cinnyris sovimanga* and *C. notatus*) and Philepittidae (*Philepitta castanea* and *Neodrepanis coruscans*) from Madagascar by PCR method

		*C. sovimanga*	*C. notatus*	*P. castanea*	*N. coruscans*
*n*		69	13	61	13
*Single*	P	2	0	6	0
	H	5	0	0	0
	L	6	1	17	3
*Double*	P, H	3	0	0	0
	P, L	5	1	6	0
	H, L	13	2	0	0
*Triple*	P, H, L	22	7	0	0
*Negative*		13	2	32	10

P, *Plasmodium* spp.; H, *Haemoproteus* spp.; L, *Leucocytozoon* spp.

Haemosporidian lineage diversity was similar in both Nectariniidae (*χ*^2^ = 1.86, d.f. = 1, *P* = 0.17) species and both Philepittidae species (*χ*^2^ = 0.27, d.f. = 1, *P* = 0.605) respectively, but differed significantly (*χ*^2^ = 5.97, d.f. = 1, *P* = 0.015) among those families (Fig. 2). Three lineages were shared between Nectariniidae and Philepittidae: pGRW09 (DQ060773), lFOMAD01 (JN032605) and lHYPMA02 (MF442609).

**Figure 3.**
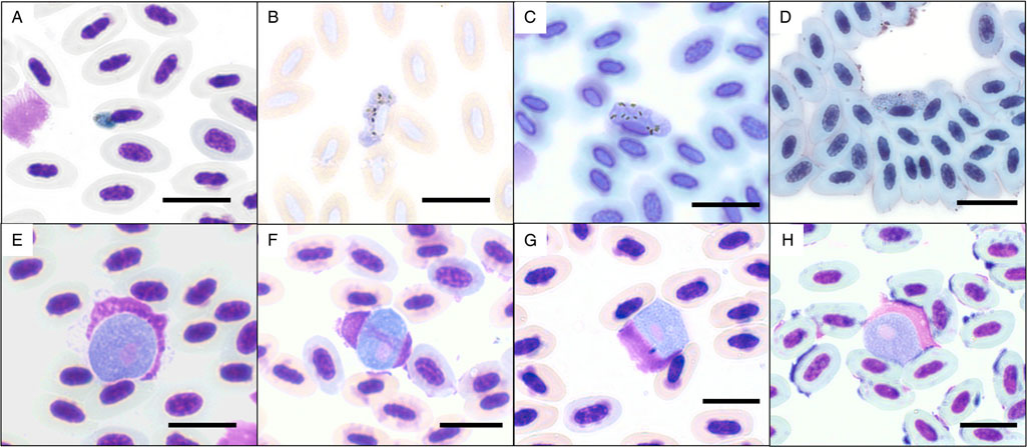
Gametocytes of haemosporidian parasites of Nectariniidae and Philepittidae from Madagascar. (a) *Plasmodium* pGRW09 from *Philepitta castanea*; (b) hNENOT04 from *Cinnyris notatus*; (c) hCINSOV01 from *C. sovimanga*; (d) hCINNOT01 from *C. notatus*; (e) lCINSOV03 from *C. sovimanga*; (f) lFOMAD01 from *C. sovimanga*; (g) lHYPMA02 from *P. castanea*; (h) lPHICAS01 from *P. castanea*. Scale bar = 10 *μ*m.

Microscopical detection of haemosporidian parasites was similar in sensitivity for Nectariniidae species (*χ*^2^ = 1.184, d.f. = 1, *P* = 0.28) but less sensitive for Philepittidae species (*χ*^2^ = 6.62, d.f. = 1, *P* = 0.01). However, in the case of mixed infections within Nectariniidae species, not all genera were equally detected. Prevalence of *Haemoproteus* spp. was similar for both detection methods (*χ*^2^ = 0.295, d.f. = 1, *P* = 0.59) while *Plasmodium* and *Leucocytozoon* species were detected significantly less often using the microscopical approach (P: *χ*^2^ = 20.62, d.f. = 1, *P* < 0.001; L: *χ*^2^ = 25.02, d.f. = 1, *P* < 0.001). Gametocytes of the lineages pGRW09, hCINSOV01, hCINNOT01, hNENOT04, lHYPMA02, lFOMAD01, lCINSOV03 and lPHICAS01 were detected in the blood smears (Fig. 3).

**Figure 4.**
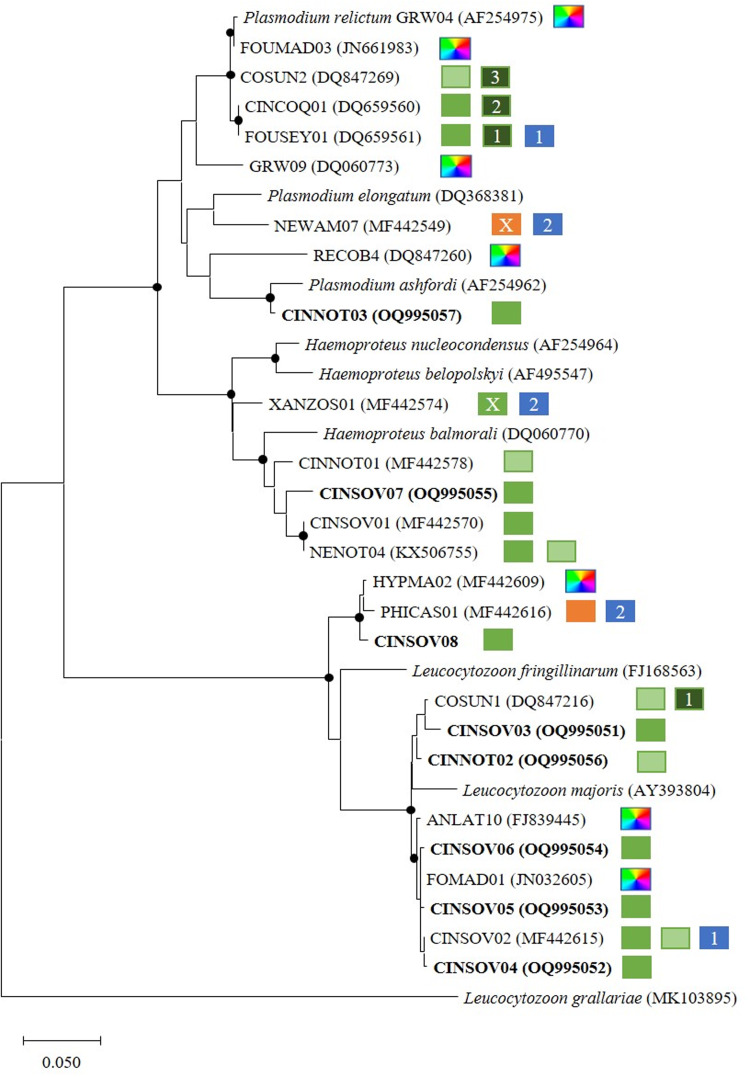
Phylogenetic relationship of mitochondrial *cytb* lineages of haemosporidian parasites (Acc. No.) detected in blood samples of Nectariniidae and Philepittidae species on Madagascar, along with sequences of different morphospecies (Acc. No.), constructed using maximum likelihood (TN93 + G). Dots on nodes indicate bootstrap values >70%. Bold text indicates new sequences. Bird hosts are indicated with colour-coded squares: dark green = *Cinnyris* spec. (*n*); green = *C. sovimanga*; light green = *C. notatus*; orange = *Philepitta castanea*; blue = other bird species (*n*) and multicoloured = >5 other bird genera. ‘X’ marks a potentially aberrant host.

Phylogenetic analyses revealed monophyly of all 3 haemosporidian genera (Fig. 4). *Plasmodium* lineages pCOSUN2 and pCINCOQ01 have been detected exclusively in *Cinnyris* species to date, as the closely related pFOUSEY01, which has additionally been detected once in *Foudia sechellarum* (Beadell *et al*., 2006). The newly found lineage pCINNOT03 differs in 8 of 479 base pairs to its most homologous lineage pMEAPI 12, which was isolated from migratory bee-eaters *Merops apiaster* (Coraciiformes) in Portugal (Emmenegger *et al*., 2020). *Haemoproteus* lineages hCINNOT01, hCINSOV07, hCINSOV01 and hNENOT04 all group together and were exclusively isolated from *C. sovimanga* and *C. notatus* so far. The *Leucocytozoon* lineages isolated during this study form 2 completely different clades. One of them consists of the generalist lineage lHYPMA02, lPHICAS01 and lCINSOV08. The other clade is again divided into 2 groups of closely related lineages. The clade consisting of lCOSUN1, lCINSOV03 and lCINNOT02 are all exclusively found in *Cinnyris* species. The other clade contains 3 newly described lineages from *C. sovimanga* (lCINSOV04-06) and lCINSOV02, along with the generalist lineages lFOMAD01 and lANLAT10.

The degree of specialization within each haemosporidian parasite lineage was determined by utilizing the host-diversity index (Hd), as indicated in Supplementary Tables 6 and 7. Notably, an Hd value of 1 was assigned to pGRW04 and pGRW09, as they are acknowledged as generalists. Table 3 included the specialization categorizations that resulted from a combination of Hd values and phylogenetic data.

**Table 3.** Predicted specialization of haemosporidian lineages (genus and lineage name) detected in Malagasy Nectariniidae (*Cinnyris sovimanga* and *C. notatus*) and Philepittidae species (*Neodrepanis coruscans* and *Philepitta castanea*)

Genus	Lineage	Bird species	Other hosts	Other host species	Site	Predicted specialization
*Plasmodium*	COSUN2	*C. notatus*	3 hosts, 1 genus	*Cinnyris cupreus*, *C. venustus*, *C. pulchellus*	Madagascar, Nigeria und Benin	Specialist
	CINCOQ01	*C. sovimanga*	3 hosts, 2 families, 2 genera	*Cinnyris notatus*, *C. coquerellii*, *Neomixis striatigula* (Cisticolidae)	Madagascar, Mayotte	Specialist
	GRW04	*P. castanea*	91 hosts, 5 order, 25 families, 62 genera		Worldwide	Generalist
	GRW09	*C. sovimanga*, *P. castanea*	88 hosts, 3 order, 22 families, 62 genera		Africa, Asia, Europe, Australia	Generalist
	FOUMAD03	*C. sovimanga*	11 hosts, 7 families, 10 genera		Madagascar	Generalist
	FOUSEY01	*C. sovimanga*	2 hosts, 2 families, 2 genera	*Cinnyris dussumieri*, *Foudia sechellarum* (Ploceidae)	Seychelles	Gpecialist
	NEWAM07	*P. castanea*	1 host, 1 family, 1 genus	*Newtonia amphicroa*		Specialist (*Newtonia amphicroa*)
	RECOB4	*C. sovimanga*	18 hosts, 4 families, 12 genera		Africa, Ecuador	Specialist (Nectariniidae)
	CINNOT03	*C. notatus*			Madagascar	Specialist
*Haemoproteus*	NENOT04	*C. notatus*, *C. sovimanga*			Madagascar	Specialist
	CINNOT01	*C. notatus*			Madagascar	Specialist
	CINSOV01	*C. sovimanga*			Madagascar	Specialist
	XANZOS01	*C. sovimanga*	3 hosts, 2 families, 2 genera	*Xanthomixis cinereiceps*, *Xanthomixis zosterops*	Madagascar	Specialist (*Xanthomixis spp.*)
	CINSOV07	*C. sovimanga*			Madagascar	Specialist
*Leucocytozoon*	FOMAD01	*C. sovimanga*	17 hosts, 7 families, 13 genera		Komoren, Mayotte, Réunion	Generalist
	COSUN1	*C. notatus*	1 host, 1 family, 1 genus	*Cinnyris cupreus*	Nigeria	Specialist
	HYPMA02	*C. sovimanga*	6 hosts, 4 families, 5 genera		Madagascar	Generalist
	ANLAT10	*C. sovimanga*	9 hosts, 2 order, 6 families, 9 genera		Gabon, Ghana, Madagascar, Italy, Sweden	Generalist
	CINSOV02	*C. notatus*, *C. sovimanga*	1 host, 1 family, 1 genus	*Hypsipetes madagascariensis* (Pycnonotidae)	Madagascar	Specialist
	CINSOV03	*C. sovimanga*			Madagascar	Specialist
	CINSOV04	*C. sovimanga*			Madagascar	Specialist
	CINSOV05	*C. sovimanga*			Madagascar	Specialist
	CINSOV06	*C. sovimanga*			Madagascar	Specialist
	CINSOV08	*C. sovimanga*			Madagascar	Specialist
	CINNOT02	*C. notatus*			Madagascar	Specialist
	PHICAS01	*Phileptta castanea*	2 hosts, 1 order, 2 families, 2 genera	*Foudia omissa* (Ploceidae), *Hypsipetes madagascariensis* (Pycnonotidae)	Madagascar	Specialist

Data of other findings are given (other hosts and sites) along with the predicted specialization of each lineage based on the host-diversity index and phylogenetic data.

**Figure 5.**
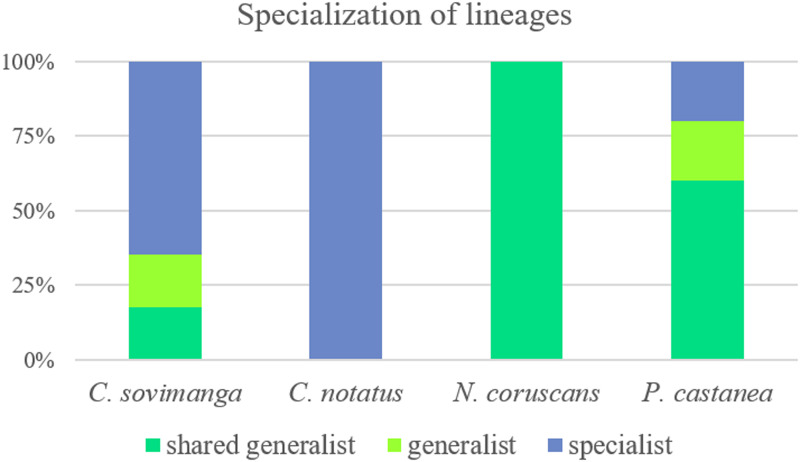
Percentage of generalist and specialist haemosporidian lineages detected in infected Nectariniidae (*Cinnyris sovimanga* and *C. notatus*) and Philepittidae species (*Neodrepanis coruscans* and *Philepitta castanea*). Generalist lineages found in both families are shown separately as ‘shared generalists’.

Infections in Nectariniidae species consist mostly of specialized haemosporidian lineages, whereas infections in Philepittidae are mainly caused by generalist lineages (Fig. 5). pNEWAM07 and hXANZOS01 are considered to be abortive infections as they seem to be specialized on other bird taxa.

### Trypanosoma abundance, prevalence and diversity

No trypanosomes were detected in blood smears during microscopic examination. Prevalence of *Trypanosoma* species detected in blood samples by molecular approach was between 7.7 and 17% in Nectariniidae and Philepittidae (Table 1). *Trypanosoma* sp. CORVOID01 was the only sequence shared between the families, once detected in *C. sovimanga* and 3 times in *P. castanea*. With 8 isolated *Trypanosoma* sequences, *C. sovimanga* exhibited the greatest diversity.

**Figure 6.**
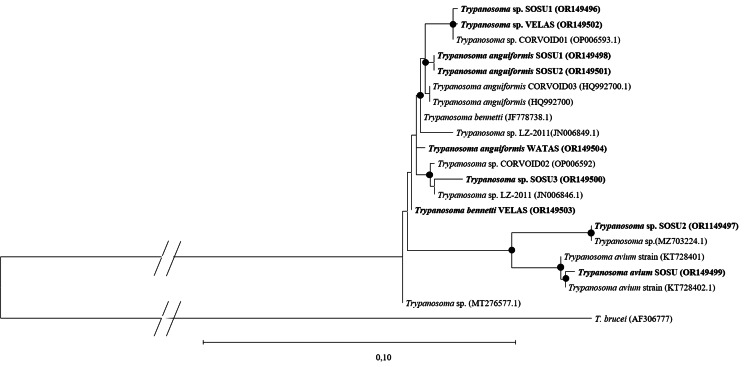
Phylogenetic relationship of *Trypanosoma* SSU rRNA sequences (Acc. No.) detected in blood samples of Nectariniidae and Philepittidae species on Madagascar, along with highly homologous sequences of *Trypanosoma* species deposited in GenBank (Acc. No.), constructed using maximum likelihood (K2 + G). Dots on nodes indicate bootstrap values >70%. Bold text indicates new sequences.

In the phylogenetic tree, *Trypanosoma* sequences detected in this study form 2 separate branches (Fig. 6). One of them contains *Trypanosoma avium* sequences, including the newly detected *T. avium* isolate SOSU, and 2 *T. avium* species sequences, one of them the isolate SOSU2. Other sequences isolated from Nectariniidae and Philepittidae in this study form the second clade, together with sequences of *T. anguiformis* and *T. bennetti.*

### Filarioid nematode abundance, prevalence and diversity

**Figure 7.**
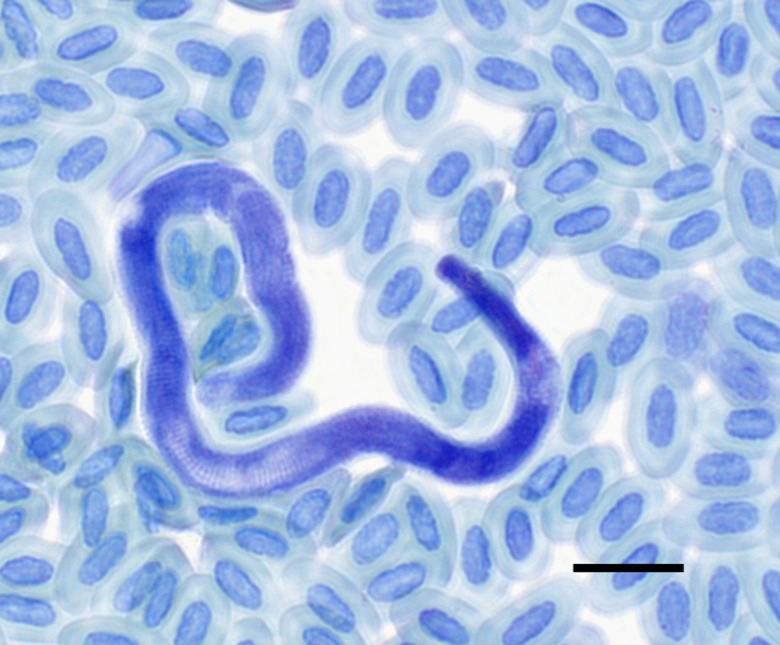
Larval stage (microfilaria) of *Splendidofilaria bartletti* isolate VELAS1 (OR148294) isolated from *Philepitta castanea* (Philepittidae) of Madagascar. Scale bar = 10 *μ*m.

Highest prevalence for filarioid nematode infection was detected in *C. notatus* (31%) using molecular detection methods (Table 1). Other examined bird taxa showed prevalence of 6–13.1%. No sequence was shared between the different bird taxa. *Splendidofilaria bartletti* VELAS1 was detected once in a blood smear of *P. castanea* during microscopic examination (Fig. 7).

**Figure 8.**
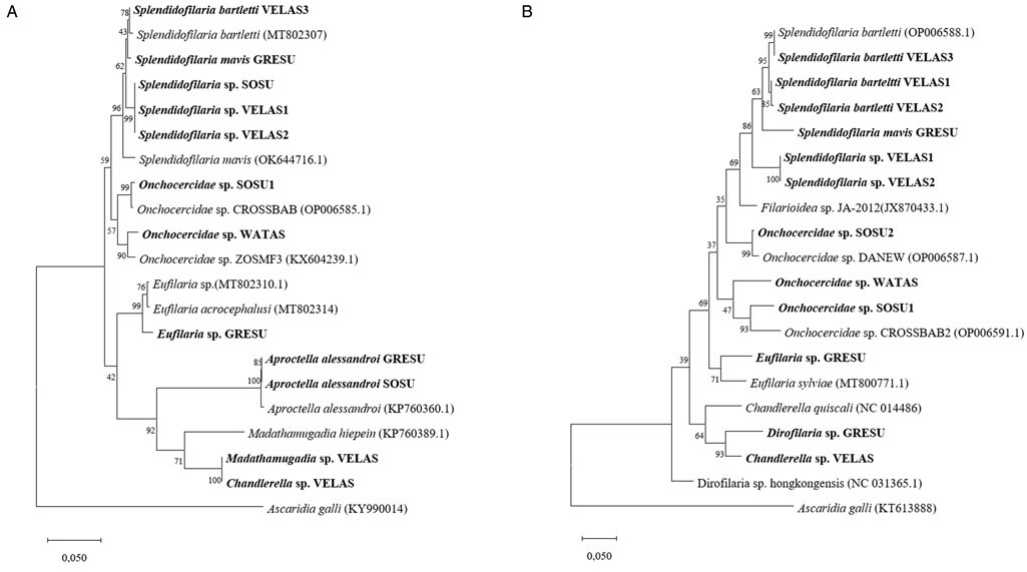
Phylogenetic relationship of filarioid nematode 28S rRNA (a) and cox1 (b) sequences detected in blood samples of Nectariniidae and Philepittidae species on Madagascar, along with highly homologous sequences of avian filarioid nematodes deposited in GenBank (Acc. No.), constructed using maximum likelihood (a: T92; b: TN93). Bootstrap values are given. Bold text indicates new sequences.

Phylogenetic examination was performed separately for 28S rRNA (Fig. 8a) and cox1 (Fig. 8b) sequences of filarioid nematodes because the comparable datasets of molecular markers are very different. In the phylogenetic tree of 28S rRNA sequences, *Aproctella alessandroi* clusters with *Madathamugadia*, and *Chandlerella* species sequences form a distinct clade. The *Eufilaria* species isolates form another clade, while *Onchocercidae* and *Splendidofilaria* species group together, forming a sister clade. This clear separation of groups cannot be seen in the phylogenetic tree using cox1 sequences of the filarioid nematodes.

### Mixed infections

Mixed infections of all blood parasite taxa were most abundant in *C. notatus* (30.7%), fewer in *C. sovimanga* (18.8%) and *P. castanea* (14.8%) and lowest in *N. coruscans* (7.7%). Single infections with haemosporidian parasites were the most common kind of infection in all avian taxa (Table 4).

**Table 4.** Kind of blood parasite infection (*n*) detected in avian blood samples of Nectariniidae (*Cinnyris sovimanga* and *C. notatus*) and Philepittidae (*Philepitta castanea* and *Neodrepanis coruscans*) from Madagascar by PCR method

		*C. sovimanga*	*C. notatus*	*P. castanea*	*N. coruscans*
*Single*	H	43	7	20	2
	T	1	0	2	0
	FN	0	0	2	1
*Double*	H, T	9	0	3	1
	H, FN	2	3	4	0
	T, FN	0	0	0	0
*Triple*	H, T, FN	2	1	2	0

H, Haemosporida.; T, *Trypanosoma*; FN, filarioid nematode.

### Molecular sexing

**Figure 9.**
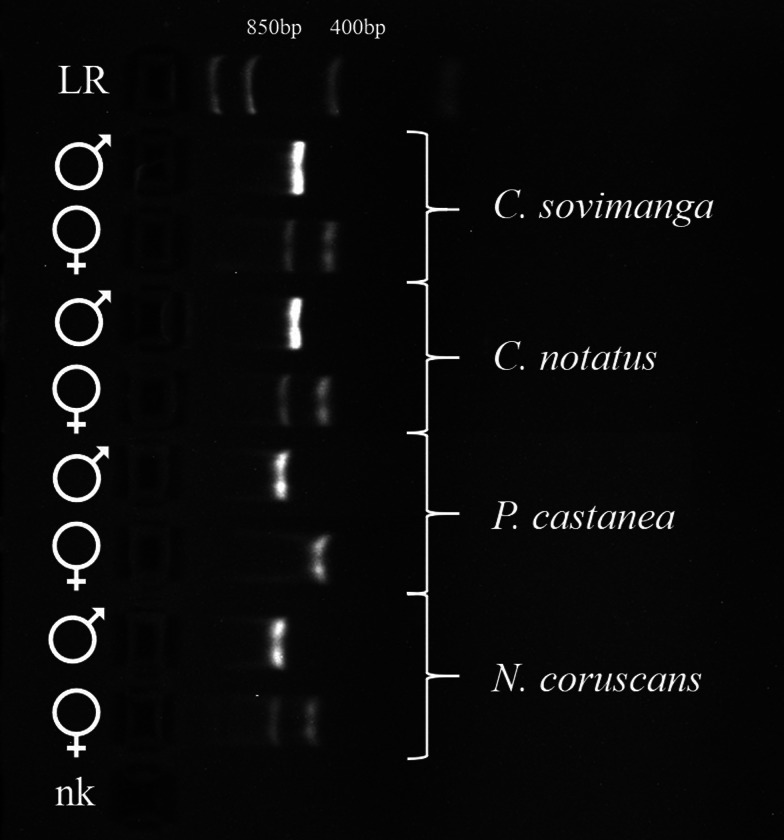
Comparison of PCR fragments in gel electrophoresis, obtained using primer set HPF/HPR (Díaz Casana *et al*., 2019). One morphologically determined male and one female of each species (*Cinnyris sovimanga* and *C. notatus* (Nectariniidae), *Philepitta castanea* and *Neodrepanis coruscans* (Philepittidae)) were included, along with a negative control. LR: FastRuler Low Range DNA Ladder (Thermofisher Scientific, Waltham, USA).

The PCR approach used for sexing gave slightly different results than expected from the original protocol by Díaz Casana *et al*. (2019). For *P. castanea* only, female bird samples showed a single band at approximately 400 bp in the agarose gel instead of 2 bands (Fig. 9). This has been approved by 7 samples of birds, which have been additionally identified as females by morphological features in the field.

### Influence of sex and age of birds on blood parasite prevalence

Statistical analyses revealed different effects of age and sex on the prevalence of haemosporidian parasites (Supplementary Tables 4 and 5). Female *C. sovimanga* tended to be infected less with *Haemoproteus* spp. than males (*χ*^2^ = 5.56, d.f. = 1, *P* = 0.018) and female Nectariniidae in general were infected less with haemosporidian parasites than males (*χ*^2^ = 4.03, d.f. = 1, *P* = 0.045). In contrast, female Philepittidae were significantly more often infected with *Leucocytozoon* spp. than males. For *Trypanosoma* species, an influence of sex was just detected in *N. coruscans* and Philepittidae (dataset of both species combined), where females were more often infected than males (*N. coruscans*: *χ*^2^ = 5.96, d.f. = 1, *P* = 0.015; Philepittidae: *χ*^2^ = 5.28, d.f. = 1, *P* = 0.022).

Haemosporidian parasites varied with the age of the different bird host families. In Nectariniidae species, juvenile birds of *C. sovimanga* were significantly less infected with each haemosporidian genus than adults and showed also lower numbers of multiple infections. *Cinnyris notatus* showed no significant differences, but combined with the results of *C. sovimanga* as Nectariniidae, differences were even more significant than with *C. sovimanga* alone. However, no significant difference was detected in Philepittidae species. For infections with *Trypanosoma* species or filarioid nematodes the age of the birds did not have a significant effect for either bird taxa.

## Discussion

The objective of this study was to investigate whether the blood parasite composition in the Malagasy common sunbird asity (*N. coruscans*) is influenced by ecological traits or by its phylogenetic background. Our initial hypothesis suggested that Nectariniidae species (*C. sovimanga* and *C. notatus*) would share similar parasite compositions and prevalence rates with *N. coruscans*, despite belonging to different avian families, due to their similarities in habitat and behavioural ecology. However, our findings did not align with this expectation. We observed significant variations in parasite prevalence and diversity between *N. coruscans* and the *Cinnyris* species. Instead, the parasite composition in *N. coruscans* appeared to be more akin to that of its close relative, *P. castanea*, particularly concerning haemosporidian parasites.

In both Nectariniidae species, a substantial number of haemosporidian lineages were detected, and there was also a high diversity of these lineages. The majority of the samples showed evidence of mixed infections, with triple infections involving all 3 genera being the most prevalent type. Specialized haemosporidian lineages appear to exist in both Malagasy sunbird species. *Neodrepanis coruscans* showed the lowest prevalence of haemosporidian infections and was only found to be infected with generalist *Leucocytozoon* lineages. *Philepitta castanea* also harboured these lineages but additionally exhibited infection with a potentially specialized *Leucocytozoon* lineage (lPHICAS01) and a few other generalist *Plasmodium* lineages. Notably, the occurrence of mixed infections was significantly lower in both Philepittidae species compared to the Nectariniidae species. The absence of triple infections involving different haemosporidian genera was attributed to the complete absence of *Haemoproteus* lineages in Philepittidae species. Given the absence of reports of generalist *Haemoproteus* species in Madagascar so far, the lack of *Haemoproteus* spp. in Philepittidae species may suggest a resistance against specialized *Haemoproteus* species within the study area. The absence of specialized haemosporidian lineages in the Philepittidae species is quite surprising. Among these lineages, lPHICAS01 appears to be the only one that might be specialized or even prefers to infect *P. castanea*, as it has been just sporadically detected in 2 other bird species (Musa *et al*., 2022). Phylogenetic analysis revealed a very close relationship (with only 4 out of 479 base pairs differing) between lPHICAS01 and the generalist lineage lHYPMA02, which was also detected in both Philepittidae species. It is possible that lPHICAS01 is in the process of developing into a specialized lineage.

The Philepittidae family of birds has ancient origins, with its roots tracing back to Asia, where it embarked on a radiation process in Madagascar over 40 million years ago (Warren *et al*., 2010). It is plausible that the ancestors of the Philepittidae were initially infected solely by generalist haemosporidian lineages that maintained their host range throughout this extended period. Interestingly, there is no evidence of speciation on the same host (with the exception of lPHICAS01) or host switching events that led to the development of specialized haemosporidian species within this family. In contrast, the Nectariniidae family arrived in Madagascar roughly 3 million years ago from Africa (Warren *et al*., 2003). It is possible that they already harboured specialized lineages, such as pCOSUN2, prior to their colonization of Madagascar. Subsequent to their arrival on the island, haemosporidian parasites within the *Cinnyris* species underwent further diversification, resulting in a broad array of specialized lineages. Phylogenetic analysis suggests that the proposed specialized lineages (lCINSOV02, lCINSOV04–lCINSOV06) may have evolved from the generalist lineage lFOMAD01. However, it remains unclear why this radiation occurred in Nectariniidae but not in Philepittidae species, which are also suitable hosts for lFOMAD01. One possible explanation is that Philepittidae species exhibit a higher tolerance to these parasites, thereby reducing the selection pressure that would drive parasite evolution towards specialization. In lineages presumed to be already specialized, as observed in Nectariniidae, there exists a certain degree of selection pressure due to coevolutionary dynamics, which favours further diversification. It would be valuable for future studies to focus on ancient bird taxa and investigate whether this observed phenomenon holds true more broadly across different avian families.

Data from 2 bird taxa at the same study site, which, unlike the species examined in this study, construct open nests, were utilized to explore the impact of nest construction on haemosporidian prevalence. In the Madagascar paradise flycatcher (*Terpsiphone mutata*), the prevalence of *Haemoproteus* and *Leucocytozoon* infection was found to be 0% (Musa *et al*., 2019), while of Vangidae showed prevalence rates in the range of 45–52% (Magaña Vázquez *et al*., 2022). Similarly, notable differences in prevalence were observed within the studied bird families that utilize closed nests (Nectariniidae exhibited a prevalence of 63–70%, whereas Philepittidae showed 0% prevalence for Haemoproteus and 31% for Leucocytozoon). Consequently, it can be concluded that the nesting behaviour of the studied bird species did not have a discernible effect on the prevalence of *Haemoproteus* and *Leucocytozoon* when compared to bird species employing open cups for nesting at the same study site.

The age of the birds had a notable impact on avian haemosporidian prevalence within the Nectariniidae family, but this influence was not observed in the Philepittidae. In adult birds, both prevalence and the occurrence of mixed infections were higher compared to juveniles, and this can be attributed to the multiplication of infections over time. Haemosporidian parasites are known to persist in bird hosts for extended periods, potentially spanning years or even a lifetime (Valkiunas, 2005). Consequently, the likelihood of being infected with haemosporidian parasites increases with the age of the host (Slowinski *et al*., 2021). Conversely, no such age-related effect was observed in Philepittidae species. This discrepancy might be attributed to the relatively low diversity of parasites within this avian taxon. Given that the pool of suitable parasites for Philepittidae appears to be limited, the maximum number of potential infections within the population may be quickly reached.

The prevalence of *Trypanosoma* and filarial nematodes exhibited similarity across different avian taxa. Neither the distinct ecological niches nor the phylogenetic backgrounds of these birds appear to result in variations in the likelihood of infection with these blood parasites. Significant differences in prevalence were not found based on the sex and age of the various bird species. While age-related differences have been documented in Phasianidae (Holmstad *et al*., 2003) and Accipitridae (Svobodová *et al*., 2015), no such variations were identified in Passeriformes, such as the pied flycatcher (Muscicapidae) (Merino and Potti, 1995). The study by Svobodová et al. (2015), which involved recaptures of Accipitridae, suggested that *Trypanosoma* infection persists throughout the lifetime of these birds. This finding elucidates the observed differences in prevalence between juveniles and adults. Conversely, since no such age-related discrepancy has been reported thus far in Passeriformes, it is reasonable to assume that *Trypanosoma* infections in these avian taxa are not of a long-lasting nature. Moreover, as there were no discernible differences in prevalence between the sexes and age groups of birds concerning microfilariae infections, it can be inferred that these infections also do not typically result in permanent, chronic infections. Additional research is needed to delve deeper into this subject.

Mixed infections involving both blood parasite taxa, *Trypanosoma* and filarioid nematodes, were frequently encountered. However, instances of double infections, where both *Trypanosoma* and filarioid nematodes were present together, were not observed. This outcome implies a potential positive correlation wherein haemosporidian infection might enhance the likelihood of acquiring infections with other blood parasite taxa. Infections with haemosporidian parasites, but not with *Trypanosoma* and filarioid nematodes, may lead to a decrease in host fitness and an increased vulnerability to other infections.

Host specialization is likely to be the case for filarioid nematodes, given the clear phylogenetic separation of their lineages and the absence of a single sequence detected in different bird species. On the other hand, the proposed generalism of avian trypanosomes (Zídková *et al*., 2012) is supported by the presence of *Trypanosoma* sp. CORVOID01 in both Nectariniidae and Philepittidae. Nevertheless, the identification of a substantial number of newly discovered *Trypanosoma* sequences exclusively in a single bird species may suggest the existence of host-specific haplotypes. However, making more precise conclusions is challenging due to limited data availability for trypanosomes and the unclear systematics in this context.

In summary, Nectariniidae and Philepittidae exhibit disparities in terms of haemosporidian parasite prevalence, diversity and specialization, while no such distinctions are apparent in the case of *Trypanosoma* sp. or filarioid nematode infections. Despite *N. coruscans* sharing similarities in habitat and behavioural ecology with both *Cinnyris* spp., it closely resembles its relative, *P.ta castanea*, in all aspects of parasitism. The initial hypothesis suggesting that nectarivorous species would display lower prevalence but higher diversity in contrast to the frugivorous *P. castanea* was not supported by our findings. Specialized *Haemoproteus* lineages have only been proposed for Nectariniidae species, as Philepittidae lack these parasites. However, specialized filarioid nematode species are detected in both avian families. Interestingly, the construction of closed nests or feeding behaviour does not appear to significantly influence the likelihood of infection with *Haemoproteus* and *Leucocytozoon* spp. in the examined bird species. Haemosporidian parasite infections in Malagasy Philepittidae and Nectariniidae appear to be primarily driven by phylogenetic factors rather than ecological ones, whereas *Trypanosoma* and filarioid nematode infections do not exhibit a clear association with either factor.

## Supporting information

Barbon et al. supplementary material Barbon et al. supplementary material

## Data Availability

The authors confirm that the data supporting the findings of this study are available within the article and its supplementary materials.

## Supplementary material

The supplementary material for this article can be found at https://doi.org/10.1017/S0031182023001075
