# Supplementary material for: Haemosporidian parasite infections of Malagasy Philepittidae and Nectariniidae are driven by phylogeny rather than ecology
Source: Parasitology. 2023 Nov 10;150(14):1316–29. doi: 10.1017/S0031182023001075 (PMC10941219; doi:10.1017/S0031182023001075)

**Supplementary Table 1:** Haemosporidian lineages (Acc. No.) detected in Malagasy Nectariniidae and Philepittidae species.

| Lineage Haemosporida (MalAvi) | Accession number (GenBank) |
| --- | --- |
| hCINNOT01 | MF442578 |
| hCINSOV01 | MF442570 |
| hCINSOV07 | OQ995055 |
| hNENOT04 | KX506755 |
| hXANZOS01 | MF442574 |
| lANLAT10 | FJ839445 |
| lCINNOT02 | OQ995056 |
| lCINSOV02 | MF442615 |
| lCINSOV03 | OQ995051 |
| lCINSOV04 | OQ995052 |
| lCINSOV05 | OQ995053 |
| lCINSOV06 | OQ995054 |
| lCINSOV08 | - |
| lCOSUN1 | DQ847216 |
| lFOMAD01 | JN032605 |
| lHYPMA02 | MF442609 |
| lPHICAS01 | MF442616 |
| pCINCOQ01 | DQ659560 |
| pCINNOT03 | OQ995057 |
| pCOSUN2 | DQ847269 |
| pFOUMAD03 | JN661983 |
| pFOUSEY01 | DQ659561 |
| pGRW04 | AF254975 |
| pGRW09 | DQ060773 |
| pNEWAM07 | MF442549 |
| pRECOB4 | DQ847260 |

**Supplementary Table 2:** *Trypanosoma* sequences (Acc. No.) isolated from Malagasy Nectariniidae and Philepittidae species.

| sequences *Trypanosoma* | Accession number (GenBank) |
| --- | --- |
| *Trypanosoma* *anguiformis* SOSU1 | OR149498 |
| *Trypanosoma* *anguiformis* SOSU2 | OR149501 |
| *Trypanosoma* *anguiformis* WATAS | OR149504 |
| *Trypanosoma* *avium* SOSU | OR149499 |
| *Trypanosoma* *bennetti* VELAS | OR149503 |
| *Trypanosoma* sp. CORVOID01 | OP006593.1 |
| *Trypanosoma* sp. LZ-2011 | JN006849.1 |
| *Trypanosoma* sp. SOSU1 | OR149496 |
| *Trypanosoma* sp. SOSU2 | OR149497 |
| *Trypanosoma* sp. SOSU3 | OR149500 |
| *Trypanosoma* sp. VELAS | OR149502 |

**Supplementary Table 3:** Mitochondrial cox1 and nuclear 28S sequences of filarioid nematodes isolated from Malagasy Nectariniidae and Philepittidae species.

| sequences filarioid nematodes | Accession number (GenBank) | |
| --- | --- | --- |
|  | **28S** | **COX1** |
| *Aproctella alessandroi* GRESU | OR159322 | - |
| *Aproctella alessandroi* SOSU | OR159324 | - |
| *Chandlerella* sp. VELAS | OR159331 | OR148300 |
| *Dirofilaria* sp. GRESU | - | OR148290 |
| *Eufilaria* sp. GRESU | OR159321 | OR148291 |
| *Madathamugadia* sp. VELAS | OR159326 |  |
| *Onchocercidae* sp. SOSU1 | OR159323 | OR148292 |
| *Onchocercidae* sp. SOSU2 | - | OR148293 |
| *Onchocercidae* sp. WATAS | OR159328 | OR148297 |
| *Splendidofilaria barteltti* VELAS1 | - | OR148294 |
| *Splendidofilaria bartletti* VELAS2 | - | OR148296 |
| *Splendidofilaria bartletti* VELAS3 | OR159330 | OR148299 |
| *Splendidofilaria mavis* GRESU | OR159320 | OR148289 |
| *Splendidofilaria* sp. SOSU | OR159325 | - |
| *Splendidofilaria* sp. VELAS1 | OR159327 | OR148295 |
| *Splendidofilaria* sp. VELAS2 | OR159329 | OR148298 |

**Supplementary Table 4:** Prevalence of blood parasites for different gender of Malagasy Nectariniidae and Philepittidae species.

| C. sovimanga |  | X= 11.15;  p< 0.001 | X= 10.871;  p< 0.001 | X= 29.048;  p< 0.001 | X= 4.481;  p= 0.034 | X= 0.195;  p= 0.656 | X= 1.081;  p= 0.298 | X= 11.152;  p< 0.001 | X= 7.684;  p= 0.006 | X= 8.221;  p= 0.004 | X= 29.048;  p< 0.001 |
| --- | --- | --- | --- | --- | --- | --- | --- | --- | --- | --- | --- |
|  | n | Haemosporida | P | H | L | Tryp | FN | koi1 | koi2 | koi3 | koi2/3 |
| juvenile | 14 | 7 | 1 | 0 | 6 | 3 | 0 | 7 | 0 | 0 | 0 |
| adult | 55 | 49 | 31 | 43 | 40 | 9 | 4 | 6 | 21 | 22 | 43 |
| C. notatus |  | X= 0.197;  p< 0.657 | X= 1.733;  p= 0.188 | X= 0.481;  p= 0.488 | X= 0.197;  p= 0.657 | X= 0.09;  p= 0.764 | X= 2.437;  p= 0.119 | X= 0.09;  p< 0.764 | X= 3.611;  p= 0.057 | X= 1.264;  p= 0.261 | X= 0.325;  p= 0.569 |
|  | n | Haemosporida | P | H | L | Tryp | FN | koi1 | koi2 | koi3 | koi2/3 |
| juvenile | 1 | 1 | 0 | 1 | 1 | 0 | 1 | 0 | 1 | 0 | 1 |
| adult | 12 | 10 | 8 | 8 | 10 | 1 | 3 | 1 | 2 | 7 | 9 |
| Nectariniidae |  | X= 9.889;  p= 0.002 | X= 13.032;  p< 0.001 | X= 25.482;  p< 0.001 | X= 4.521;  p= 0.033 | X= 0.237;  p= 0.626 | X= 0.199;  p= 0.656 | X= 11.356;  p< 0.001 | X= 4.53;  p= 0.033 | X= 10.045;  p< 0.001 | X= 26.987;  p< 0.001 |
|  | n | Haemosporida | P | H | L | Tryp | FN | koi1 | koi2 | koi3 | koi2/3 |
| juvenile | 15 | 8 | 1 | 1 | 7 | 3 | 1 | 7 | 1 | 0 | 1 |
| adult | 67 | 59 | 39 | 51 | 50 | 10 | 7 | 7 | 23 | 29 | 52 |
| P. castanea |  | X= 3.36;  p= 0.057 | X= 1.714;  p= 0.19 | X= 0;  p= 1 | X= 1.948;  p= 0.163 | X= 0.164;  p= 0.686 | X= 0.587;  p= 0.444 | X= 1.948;  p= 0.163 | X= 0.75;  p= 0.386 | X= 0;  p= 1 | X= 0.75;  p= 0.386 |
|  | n | Haemosporida | P | H | L | Tryp | FN | koi1 | koi2 | koi3 | koi2/3 |
| juvenile | 27 | 10 | 4 | 0 | 8 | 4 | 5 | 8 | 2 | 0 | 2 |
| adult | 27 | 17 | 8 | 0 | 13 | 3 | 3 | 13 | 4 | 0 | 4 |
| N. coruscans |  | X= 3.176;  p= 0.075 | X= 0;  p= 1 | X= 0;  p= 1 | X= 3.176;  p= 0.075 | X= 1.019;  p= 0.313 | X= 1.019;  p= 0.313 | X= 3.176;  p= 0.075 | X= 2.077;  p= 0.15 | X= 0;  p= 1 | X= 2.077;  p= 0.15 |
|  | n | Haemosporida | P | H | L | Tryp | FN | koi1 | koi2 | koi3 | koi2/3 |
| juvenile | 4 | 0 | 0 | 0 | 0 | 0 | 0 | 0 | 0 | 0 | 0 |
| adult | 9 | 3 | 0 | 0 | 3 | 1 | 1 | 3 | 2 | 0 | 2 |
| Philepittidae |  | X= 3.656;  p= 0.056 | X= 0.984;  p= 0.321 | X= 0;  p= 1 | X= 2.517;  p= 0.183 | X= 0.051;  p= 0.821 | X= 0.361;  p= 0.548 | X= 2.513  p= 0.113 | X= 1.653;  p= 0.199 | X= 0;  p= 1 | X= 2.348;  p= 0.125 |
|  | n | Haemosporida | P | H | L | Tryp | FN | koi1 | koi2 | koi3 | koi2/3 |
| juvenile | 31 | 10 | 4 | 0 | 8 | 4 | 5 | 8 | 2 | 0 | 2 |
| adult | 36 | 20 | 8 | 0 | 16 | 4 | 4 | 16 | 6 | 0 | 6 |

**Supplementary Table 5:** Prevalence of blood parasites for different age of Malagasy Nectariniidae and Philepittidae species.

| C. sovimanga |  | X= 2.717;  p= 0.099 | X= 0.017;  p= 0.896 | X= 5.558;  p= 0.018 | X= 0.336;  p= 0.562 | X= 0.031;  p= 0.86 | X= 0.009;  p= 0.924 | X= 1.294;  p= 0.264 | X= 1.145;  p= 0.285 | X= 1.732;  p=0.188 | X= 0.067;  p= 0.796 |
| --- | --- | --- | --- | --- | --- | --- | --- | --- | --- | --- | --- |
|  | n | Haemosporida | P | H | L | Tryp | FN | koi1 | koi2 | koi3 | koi2/3 |
| f | 34 | 27 | 17 | 18 | 23 | 6 | 2 | 5 | 13 | 9 | 22 |
| m | 31 | 29 | 15 | 25 | 23 | 6 | 2 | 8 | 8 | 13 | 21 |
| C. notatus |  | X= 1.477;  p= 0.224 | X= 1.17;  p= 0.279 | X= 0.325;  p= 0.569 | X= 1.477;  p= 0.224 | X= 1.737;  p= 0.188 | X= 0.325;  p= 0.569 | X= 1.733;  p= 0.188 | X= 0.043;  p= 0.836 | X= 0.124;  p=0.725 | X= 0.043;  p= 0.836 |
|  | n | Haemosporida | P | H | L | Tryp | FN | koi1 | koi2 | koi3 | koi2/3 |
| f | 8 | 6 | 4 | 6 | 6 | 0 | 2 | 0 | 2 | 4 | 6 |
| m | 5 | 5 | 4 | 3 | 5 | 1 | 2 | 1 | 1 | 3 | 4 |
| Nectariniidae |  | X= 4.032;  p= 0.045 | X= 0.06;  p= 0.806 | X= 3.714;  p= 0.054 | X= 0.751;  p= 0.386 | X= 0.371;  p= 0.542 | X= 0.053;  p= 0.818 | X= 2.257;  p= 0.133 | X= 1.045;  p= 0.307 | X= 1.511;  p=0.219 | X= 0.069;  p= 0.793 |
|  | n | Haemosporida | P | H | L | Tryp | FN | koi1 | koi2 | koi3 | koi2/3 |
| f | 42 | 33 | 21 | 24 | 29 | 6 | 4 | 5 | 15 | 13 | 28 |
| m | 36 | 34 | 19 | 28 | 28 | 7 | 4 | 9 | 9 | 16 | 25 |
| P. castanea |  | X= 0.774;  p= 0.379 | X= 1.787;  p= 0.181 | X= 0;  p= 1 | X= 3.458;  p= 0.063 | X= 2.371;  p= 0.124 | X= 0.071;  p= 0.789 | X= 1.08;  p= 0.299 | X= 0.061;  p= 0.805 | X= 0;  p= 1 | X= 0.061;  p= 0.805 |
|  | n | Haemosporida | P | H | L | Tryp | FN | koi1 | koi2 | koi3 | koi2/3 |
| f | 13 | 8 | 1 | 0 | 8 | 3 | 2 | 7 | 1 | 0 | 1 |
| m | 40 | 19 | 10 | 0 | 13 | 3 | 5 | 15 | 4 | 0 | 4 |
| N. coruscans |  | X= 0.965;  p= 0.326 | X= 0;  p= 1 | X= 0;  p= 1 | X= 0.965;  p= 0.326 | X= 5.958;  p= 0.015 | X= 0.197;  p= 0.657 | X= 0.965;  p= 0.326 | X= 0;  p= 1 | X= 0;  p= 1 | X= 0;  p= 1 |
|  | n | Haemosporida | P | H | L | Tryp | FN | koi1 | koi2 | koi3 | koi2/3 |
| f | 2 | 1 | 0 | 0 | 1 | 1 | 0 | 1 | 0 | 0 | 0 |
| m | 11 | 2 | 0 | 0 | 2 | 0 | 1 | 2 | 0 | 0 | 0 |
| Philepittidae |  | X= 1.656;  p= 0.198 | X= 1.398;  p= 0-237 | X= 0;  p= 1 | X= 4.687;  p= 0.03 | X= 5.281.;  p= 0.022 | X= 0.027;  p= 0.869 | X= 1.97;  p= 0.16 | X= 0.023;  p= 0.879 | X= 0;  p= 1 | X= 0.023;  p= 0.879 |
|  | n | Haemosporida | P | H | L | Tryp | FN | koi1 | koi2 | koi3 | koi2/3 |
| f | 15 | 9 | 1 | 0 | 9 | 4 | 2 | 8 | 1 | 0 | 1 |
| m | 51 | 21 | 10 | 0 | 15 | 3 | 6 | 17 | 4 | 0 | 4 |

**Supplementary Table 6:** Host table of lineages detected in Malagasy Nectariniidae and Philepittidae. Detailed information about host species, genus, family total n and Reference are given. Additional comments are shown under “notes”.

| genus | lineage | host species | genus | family | n | Reference | note |
| --- | --- | --- | --- | --- | --- | --- | --- |
| Plasmodium | COSUN2 | *Cinnyris cupreus* | *Cinnyris* | Nectariniidae | 1 | Hellgren *et al*. 2017 | P-COSUN1 |
|  |  | *Cinnyris venustus* | *Cinnyris* | Nectariniidae | 1 | Hellgren *et al*. 2017 | P-COSUN1 |
|  |  | *Cinnyris notatus* | *Cinnyris* | Nectariniidae | 2 | Musa *et al*. 2019 |  |
|  |  | *Cinnyris pulchellus* | *Cinnyris* | Nectariniidae | 2 | Harvey & Voelkel, 2017 | n unclear |
| Plasmodium | CINCOQ01 | *Cinnyris sovimanga* | *Cinnyris* | Nectariniidae | 19 | Ishtiaq *et al*., 2012; Lauron *et al*., 2014; Musa *et al*., 2023 | PV20 |
|  |  | *Cinnyris notatus* | *Cinnyris* | Nectariniidae | 2 |  | P20 |
|  |  | *Cinnyris coquerellii* | *Cinnyris* | Nectariniidae | 3 | Beadell *et al*., 2006; Ishtiaq *et al*., 2021 | cited each other |
|  |  | *Neomixis striatigula* | *Neomixis* | Cisticolidae | 2 | Musa *et al*. 2019 |  |
| Plasmodium | GRW04 | *91 hosts* | *62 genera* | 25 families |  | Musa *et al*., 2023 |  |
| Plasmodium | GRW09 | *88 hosts* | *62 genera* | 22 families |  | Musa *et al*., 2023 |  |
| Plasmodium | FOUMAD03 | *Cinnyris sovimanga* | *Cinnyris* | Nectariniidae | 1 | Musa *et al*., 2023 |  |
|  |  | *Foudia madagascariensis* | *Foudia* | Ploceidae | 4 | Ishtiaq *et al*., 2012; Musa *et al*., 2019; Musa *et al*., 2022 | n unclear |
|  |  | *Foudia omissa* | *Foudia* | Ploceidae | 5 | Musa *et al*., 2019; Musa *et al*., 2022 |  |
|  |  | *Ploceus nelicourvi* | *Ploceus* | Ploceidae | 1 | Musa *et al*., 2019 |  |
|  |  | *Acrocephalus newtoni* | *Acrocephalus* | Acrocephalidae | 1 | Musa *et al*. 2019 |  |
|  |  | *Nesillas typica* | *Nesillas* | Acrocephalidae | 9 | Musa *et al*., 2019 |  |
|  |  | *Bernieria madagascariensis* | *Bernieria* | Bernieridae | 1 | Musa *et al*., 2019 |  |
|  |  | *Xanthomixis zosterops* | *Xanthomixis* | Bernieridae | 4 | Musa *et al*., 2019 |  |
|  |  | *Copsychus albospecularis* | *Copsychus* | Muscicapidae | 1 | Musa *et al*., 2019 |  |
|  |  | *Saxicola torquatus* | *Saxicola* | Muscicapidae | 2 | Musa *et al*., 2019 |  |
|  |  | *Cyanolanius madagascarinus* | *Cyanolanis* | Vangidae | 2 | Musa *et al*., 2019; Magaña Vazquez *et al*., 2022 |  |
| Plasmodium | FOUSEY01 | *Cinnyris sovimanga* | *Cinnyris* | Nectariniidae | 1 | Musa *et al*., 2023 |  |
|  |  | *Cinnyris dussumieri* | *Cinnyris* | Nectariniidae | 1 | Beadell *et al*., 2006 |  |
|  |  | *Foudia sechellarum* | *Foudia* | Ploceidae | 1 | Beadell *et al*., 2006 |  |
| Plasmodium | NEWAM07 | *Philepitta castanea* | *Philepitta* | Philepittidae | 1 | Musa *et al*., 2023 |  |
|  |  | *Newtonia amphicroa* | *Newtonia* | Vangidae | 5 | Musa *et al*., 2019; Magaña Vazquez *et al*., 2022 |  |
|  |  | *Vanga curvirostris* | *Vanga* | Vangidae | 1 | Magaña Vazquez *et al*., 2022 |  |
|  |  | *Xenopirostris polleni* | *Xenopirostris* | Vangidae | 1 | Magaña Vazquez *et al*., 2022 |  |
| Plasmodium | RECOB4 | *Cinnyris sovimanga* | *Cinnyris* | Nectariniidae | 1 | Musa *et al*., 2023 |  |
|  |  | *Cinnyris bouvieri* | *Cinnyris* | Nectariniidae | 1 | Lauron *et al*., 2014 |  |
|  |  | *Cinnyris reichnowi preussi* | *Cinnyris* | Nectariniidae | 3 | Lauron *et al*., 2014 |  |
|  |  | *Cinnyris venustus* | *Cinnyris* | Nectariniidae | 1 | Lauron *et al*., 2014 |  |
|  |  | *Cinnyris chalybeus* | *Cinnyris* | Nectariniidae | 2 | Lauron *et al*., 2014 |  |
|  |  | *Cinnyris mediocris fuelleborni* | *Cinnyris* | Nectariniidae | 1 | Lauron *et al*., 2014 |  |
|  |  | *Nectarinia afra /Cinnyris afer* | *Cinnyris* | Nectariniidae | 1 | Lauron *et al*., 2014 |  |
|  |  | *Cinnyris cupreus* | *Cinnyris* | Nectariniidae | 2 | Harvey & Voelkel, 2019 | n unclear |
|  |  | *Hedydipna collaris* | *Hedydipna* | Nectariniidae | 9 | Harvey & Voelkel, 2020; Lauron *et al*., 2014 | n unclear |
|  |  | *Cyanomitra olivacea* | *Cyanomitra* | Nectariniidae | 19 | Lauron *et al*., 2014; Beadell *et al*., 2009 | n unclear by Beadell |
|  |  | *Cyanomitra verticalis* | *Cyanomitra* | Nectariniidae | 11 | Lauron *et al*., 2014; Beadell *et al*., 2009 | n unclear by Beadell |
|  |  | *Cyanomitra cyanolema* | *Cyanomitra* | Nectariniidae | 1 | Beadell *et al*., 2009 |  |
|  |  | *Chalcomitra amethystina* | *Chalcomitra* | Nectariniidae | 1 | Lauron *et al*., 2014 |  |
|  |  | *Chalcomitra senegalensis* | *Chalcomitra* | Nectariniidae | 2 | Harvey & Voelkel, 2018 | n unclear |
|  |  | *Anabathmis newtonii* | *Anabathmis* | Nectariniidae | 1 | Lauron *et al*., 2014 |  |
|  |  | *Anthreptes seimundi* | *Anthreptes* | Nectariniidae | 1 | Beadell *et al*., 2009 |  |
|  |  | *Deleornis fraseri* | *Deleornis* | Nectariniidae | 2 | Beadell *et al*., 2009 |  |
|  |  |  |  | Nectariniidae | 13 | Loiseau *et al*., 2017 | species unclear |
|  |  |  |  | Estrildidae | 2 | Loiseau *et al*., 2017 | species unclear |
|  |  | *Terpsiphone rufiventer* | *Terpsiphone* | Monarchidae | 1 | Harvey & Voelkel, 2017 |  |
| Plasmodium | CINNOT03 | *Cinnyris notatus* | *Cinnyris* | Nectariniidae | 1 | Musa *et al*., 2023 |  |
|  |  |  |  |  |  |  |  |
| Haemoproteus | CINNOT01 | *Cinnyris notatus* | *Cinnyris* | Nectariniidae | 2 | Musa *et al*., 2023 |  |
| Haemoproteus | NENOT04 | *Cinnyris notatus* | *Cinnyris* | Nectariniidae | 5 | Musa *et al*., 2023 |  |
|  |  | *Cinnyris sovimanga* | *Cinnyris* | Nectariniidae | 4 | Musa *et al*., 2023 |  |
| Haemoproteus | CINSOV01 | *Cinnyris sovimanga* | *Cinnyris* | Nectariniidae | 28 | Musa *et al*., 2023 |  |
| Haemoproteus | XANZOS01 | *Cinnyris sovimanga* | *Cinnyris* | Nectariniidae | 1 | Musa *et al*., 2023 |  |
|  |  | *Xanthomixis cinereiceps* | *Xanthomixis* | Bernieridae | 2 | Musa *et al*., 2019 |  |
|  |  | *Xanthomixis zosterops* | *Xanthomixis* | Bernieridae | 5 | Musa *et al*., 2019 |  |
| Haemoproteus | CINSOV07 | *Cinnyris sovimanga* | *Cinnyris* | Nectariniidae | 1 | Musa *et al*., 2023 |  |
|  |  |  |  |  |  |  |  |
| Leucocytozoon | FOMAD01 | *Foudia madagascariensis* | *Foudia* | Ploceidae | 5 | Cornuault *et al*., 2012; Musa *et al*., 2022 |  |
|  |  | *Foudia omissa* | *Foudia* | Ploceidae | 16 | Musa *et al*., 2022; Musa *et al*., 2019 |  |
|  |  | *Cinnyris sovimanga* | *Cinnyris* | Nectariniidae | 23 | Musa *et al*., 2023 |  |
|  |  | *Neodrepanis coruscans* | *Neodrepanis* | Philepittidae | 1 | Musa *et al*., 2023 |  |
|  |  | *Philepitta castanea* | *Philepitta* | Philepittidae | 4 | Musa *et al*., 2023 |  |
|  |  | *Hypsipetes madagascariensis* | *Hypsipetes* | Pycnonotidae | 11 | Musa *et al*., 2019 |  |
|  |  | *Nesillas typica* | *Nesillas* | Acrocephalidae | 1 | Musa *et al*., 2019 |  |
|  |  | *Bernieria madagascariensis* | *Bernieria* | Bernieridae | 2 | Musa *et al*., 2019 |  |
|  |  | *Oxylabes madagascariensis* | *Oxylabes* | Bernieridae | 2 | Musa *et al*., 2019 |  |
|  |  | *Newtonia amphichroa* | *Newtonia* | Vangidae | 1 | Magaña Vazquez *et al*., 2022 |  |
|  |  |  |  |  |  |  |  |
| Leucocytozoon | COSUN1 | *Cinnyris notatus* | *Cinnyris* | Nectariniidae | 7 | Musa *et al*., 2023 |  |
|  |  | *Cinnyris cupreus* | *Cinnyris* | Nectariniidae | 1 | Hellgren *et al*., 2007 |  |
| Leucocytozoon | HYPMA02 | *Cinnyris sovimanga* | *Cinnyris* | Nectariniidae | 2 | Musa *et al*., 2023 |  |
|  |  | *Neodrepanis coruscans* | *Neodrepanis* | Philepittidae | 2 | Musa *et al*., 2023 |  |
|  |  | *Philepitta castanea* | *Philepitta* | Philepittidae | 10 | Musa *et al*., 2023 |  |
|  |  | *Foudia omissa* | *Foudia* | Ploceidae | 56 | Musa *et al*., 2022 |  |
|  |  | *Foudia madagascariensis* | *Foudia* | Ploceidae | 6 | Musa *et al*., 2022 |  |
|  |  | *Calicalicus madagascariensis* | *Calicalicus* | Vangidae | 1 | Magaña Vazquez *et al*., 2022 |  |
| Leucocytozoon | ANLAT10 | *Cinnyris sovimanga* | *Cinnyris* | Nectariniidae | 1 | Musa *et al*., 2023 |  |
|  |  | *Andropadus latirostris* | *Andropadus* | Pycnonotidae | 1 | Valkiunas *et al*., 2009 |  |
|  |  | *?* | *?* | Pycnonotidae | 1 | Loiseau *et al*., 2017 |  |
|  |  | *Ispidina picta* | *Ispidina* | Alcedinidae | 1 | Loiseau *et al*., 2017 |  |
|  |  | *Euplectes hodeaceus* | *Euplectes* | Ploceidae | 1 | Loiseau *et al*., 2017 |  |
|  |  | *Ploceus nigricollis* | *Ploceus* | Ploceidae | 1 | Loiseau *et al*., 2018 |  |
| Leucocytozoon | CINSOV02 | *Cinnyris notatus* | *Cinnyris* | Nectariniidae | 1 | Musa *et al*., 2023 |  |
|  |  | *Cinnyris sovimanga* | *Cinnyris* | Nectariniidae | 2 | Musa *et al*., 2023 |  |
|  |  | *Hypsipetes madagascariensis* | *Hypsipetes* | Pycnonotidae | 1 | Musa *et al*. 2022 |  |
|  |  | *Terpsiphone mutata* | *Terpsiphone* | Monarchidae | 1 | Magaña Vazquez *et al*., 2022 |  |
| Leucocytozoon | CINSOV03 | *Cinnyris sovimanga* | *Cinnyris* | Nectariniidae | 5 | Musa *et al*., 2023 |  |
| Leucocytozoon | CINSOV04 | *Cinnyris sovimanga* | *Cinnyris* | Nectariniidae | 1 | Musa *et al*., 2023 |  |
| Leucocytozoon | CINSOV05 | *Cinnyris sovimanga* | *Cinnyris* | Nectariniidae | 2 | Musa *et al*., 2023 |  |
| Leucocytozoon | CINSOV06 | *Cinnyris sovimanga* | *Cinnyris* | Nectariniidae | 1 | Musa *et al*., 2023 |  |
| Leucocytozoon | CINSOV08 | *Cinnyris sovimanga* | *Cinnyris* | Nectariniidae | 1 | Musa *et al*., 2023 |  |
| Leucocytozoon | CINNOT02 | *Cinnyris notatus* | *Cinnyris* | Nectariniidae | 1 | Musa *et al*., 2023 |  |
| Leucocytozoon | PHICAS01 | *Philepitta castanea* | *Philepitta* | Philepittidae | 6 | Musa *et al*., 2023 |  |
|  |  | *Foudia omissa* | *Foudia* | Ploceidae | 2 | Musa *et al*., 2022 |  |
|  |  | *Hypsipetes madagascariensis* | *Hypsipetes* | Pycnonotidae | 1 | Musa *et al*., 2022 |  |

**Supplementary Table 7:** Host-Diversity Index given for species (HdS), genus (HdG) and family level (HdF) of bird hosts known of haemosporidian lineages detected in Malagasy Nectariniidae and Philepittidae. Predicted specialization according to the formula is given, along with the degree of specialization proposed after combining Hd values and phylogenetic data (Musa *et al*. 2023). Additional comments are shown under “notes”.

| genus | lineage | HdS | HdG | HdF | Musa *et al*., 2023 | Formula from Musa *et al*., 2019 | note |
| --- | --- | --- | --- | --- | --- | --- | --- |
| *Plasmodium* | COSUN2 | 0.9 | 0 | 0 | specialist | Family and genus specialist, species generalist |  |
|  | CINCOQ1 | 0.42 | 0.2 | 0.2 | specialist | specialist |  |
|  | GRW04 | 1 | 1 | 1 | generalist | generalist * | Determined according to previous literature |
|  | GRW09 | 1 | 1 | 1 | generalist | generalist* | Determined according to previous literature |
|  | FOUMAD03 | 0.9 | 0.82 | 0.78 | generalist | generalist |  |
|  | FOUSEY01 | 1 | 0.67 | 0.67 | specialist | generalist * | According to formula, would be considered a generalist, however, phylogeny states otherwise (phylogenetic similar to CINCOQ01, which is a specialist lineage |
|  | NEWAM07 | 0.64 | 0.64 | 0.25 | specialist | generalist* | According to formula, would be considered a generalist, however, previous studies (Musa *et al*., 2022) suppose specialization on Vangidae |
|  | RECOB4 | 0.85 | 0.68 | 0.08 | specialist | Species and genera generalist, family specialist |  |
|  | CINNOT03 | 0 | 0 | 0 | specialist | specialist |  |
|  |  |  |  |  |  |  |  |
| *Haemoproteus* | CINNOT01 | 0 | 0 | 0 | specialist | specialist |  |
|  | NENOT04 | 0.55 | 0 | 0 | specialist | Family and genera specialist, species generalist |  |
|  | CINSOV01 | 0 | 0 | 0 | specialist | specialist |  |
|  | XANZOS01 | 0.61 | 0.25 | 0.25 | specialist | Family and genera specialist, species generalist |  |
|  | CINSOV07 | 0 | 0 | 0 | specialist | specialist |  |
|  |  |  |  |  |  |  |  |
| *Leucocytozoon* | FOMAD01 | 0.8 | 0.75 | 0.75 | generalist | generalist |  |
|  | COSUN1 | 0 | 0 | 0 | specialist | specialist |  |
|  | HYPMA02 | 0.45 | 0.34 | 0.33 | generalist | specialist | According to formula, would be considered a specialist, however, previous studies prove generalist tendencies |
|  | ANLAT10 | 1 | 1 | 0.87 | generalist | generalist |  |
|  | CINSOV02 | 0.9 | 0.7 | 0.7 | specialist | generalist | According to formula, would be considered a generalist, however, supposed specialization on Cinnyris spp. |
|  | CINSOV03 | 0 | 0 | 0 | specialist | specialist |  |
|  | CINSOV04 | 0 | 0 | 0 | specialist | specialist |  |
|  | CINSOV05 | 0 | 0 | 0 | specialist | specialist |  |
|  | CINSOV06 | 0 | 0 | 0 | specialist | specialist |  |
|  | CINSOV08 | 0 | 0 | 0 | specialist | specialist |  |
|  | CINNOT02 | 0 | 0 | 0 | specialist | specialist |  |
|  | PHICAS01 | 0.56 | 0.56 | 0.56 | specialist | unclear | According to formula, unclear specialization, however, supposed ongoing specialization on Philepitta castanea |

**Supplementary Figure 1:** Rarefaction curve of bird blood samples collected from Malagasy Philepittidae and Nectariniidae in the Maromizaha rainforest, Madagascar.


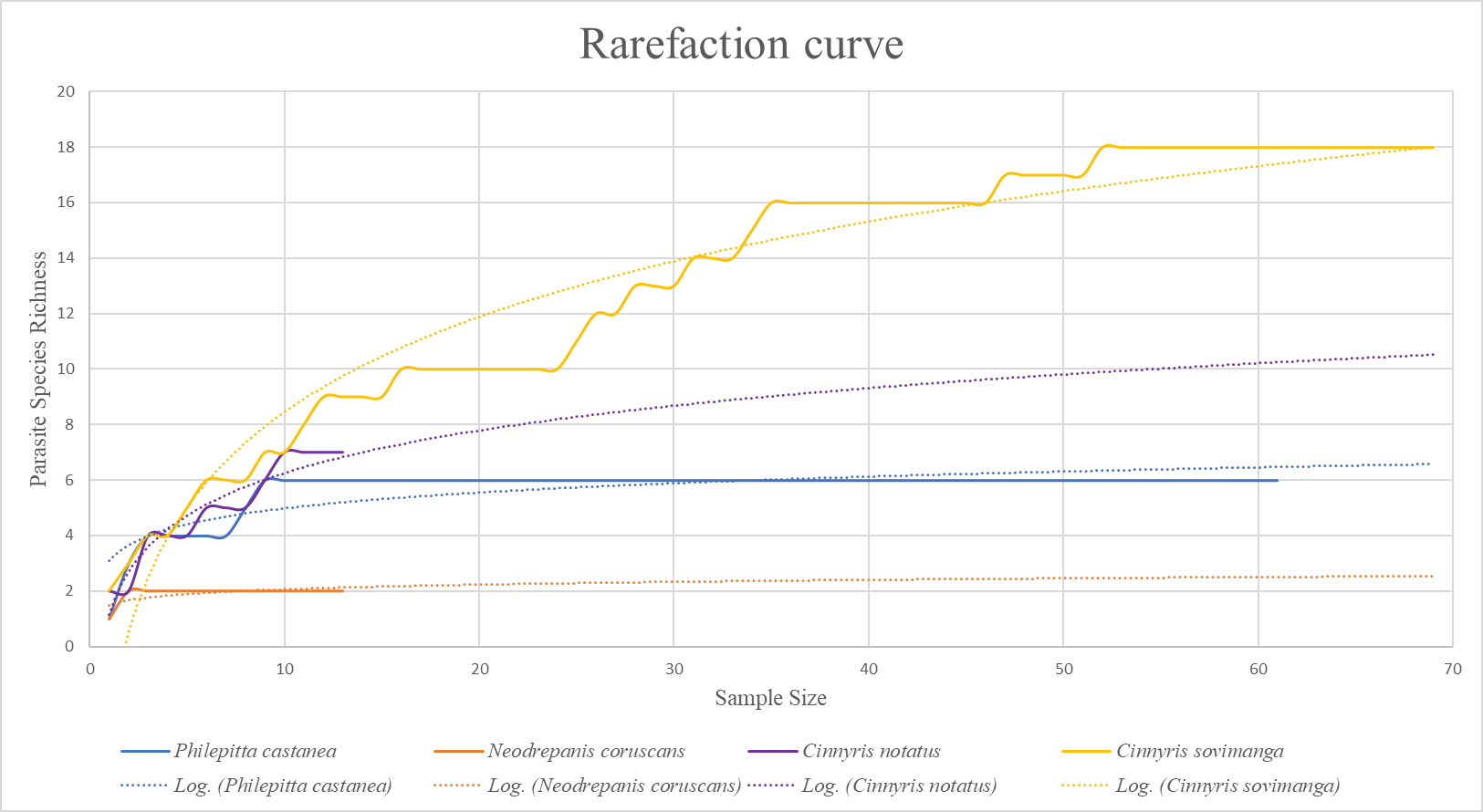

Supplement: Barbon et al. supplementary material [file S0031182023001075sup001.docx]
